# Research on seedling grading of *Platycladus orientalis* based on fuzzy C-means clustering algorithm improved by genetic sparrow family search optimization

**DOI:** 10.3389/fpls.2026.1929618

**Published:** 2026-09-03

**Authors:** Yang Liu, Juan Zhou, Juan Wang, Bao Di, Chao Wang, ZhenHui Ren

**Affiliations:** 1 Department of Computer Application Engineering, Hebei Software Institute, Baoding, China; 2 College of Mechanical and Electrical Engineering, Hebei Agricultural University, Baoding, China; 3 College of Horticulture, Hebei Agricultural University, Baoding, China; 4 College of Information Science and Technology, Hebei Agricultural University, Baoding, China

**Keywords:** EIS (electrical impedance spectroscopy), FCM (fuzzy C-means), GSFS-FCM (genetic sparrow family search - fuzzy C-means), P. orientalis, SSA (sparrow search algorithm)

## Abstract

*Platycladus orientalis* seedlings possess significant ecological and economic value, but large individual quality variation often leads to poor post-transplant performance. Existing grading standards are oversimplified with ambiguous boundaries. In this study, eight-month-old *P. orientalis* seedlings were evaluated using *in situ* electrical impedance spectroscopy (EIS) for non-destructive root assessment, combined with three aboveground morphological parameters (stem diameter, plant height, branch number). These five indices served as reliable quality predictors. To overcome the limitations of conventional clustering methods-namely, the need to pre-specify the number of clusters and sensitivity to initial centres-we proposed a modified fuzzy C-means algorithm optimised by a genetic sparrow family search (GSFS, an improved version of the sparrow search algorithm, SSA), denoted as GSFS-FCM, which autonomously determines the optimal number of quality grades. The GSFS-FCM algorithm showed that six clusters minimised the loss function. From a practical production perspective, merging Clusters 4 and 5 into a single grade yields a five-grade classification that maintains biological utility and aligns with nursery practices. Compared with traditional methods, this five-grade system significantly enhanced post-transplant survival and growth performance, with intra-grade survival rates ranging from 85.0% to 97.8% (SE  1.2–2.1%) and clearly distinguishable inter-grade differences. This approach improves the scientific rigor of *P. orientalis* seedling grading and provides a novel framework for quality classification of other woody plant seedlings.

## 1 Introduction

*P. orientalis*, an evergreen coniferous tree belonging to the family Cupressaceae, serves as a key afforestation and greening species in China. Its prominence stems from its low water requirements, strong adaptability to diverse planting conditions, and high-quality timber properties (Zhang et al., 2017; Li et al., 2023). The annual demand for *P. orientalis* seedlings in China approximates 2 billion, with seedlings typically cultivated in nurseries for 2–3 years prior to outplanting. However, substantial quality variability exists among *P. orientalis* seedlings, which significantly impair post-transplant survival rates and subsequent growth performance (Dou et al., 2024). Given that seedling quality directly dictates long-term development and timber yield, the establishment of a scientific grading system for *P. orientalis* seedlings has remained a persistent challenge in practical production and garnered considerable research attention (Chang et al., 2023).

Currently, the quality grading of *P. orientalis* seedlings primarily relies on simple above-ground morphological parameters (e.g., seedling height and stem diameter), supplemented by a limited number of easily measurable root morphological indicators (Ou et al., 2018). In contrast, more critical root parameters-such as total root length and root surface area-require destructive measurements using root scanners, a process that is both time-consuming and labor-intensive (Liu et al., 2019). Furthermore, the sampling procedure inevitably disturbs and damages the root’s native soil environment, potentially compromising the reliability and accuracy of the results. In recent years, various root detection techniques—including minirhizotron systems, ground-penetrating radar, nuclear magnetic resonance imaging, isotopic tracing, X-ray scanning analysis, and high-density electrical resistivity methods—have advanced rapidly (Liu et al., 2019). Nevertheless, each of these techniques possesses inherent limitations. Electrical impedance spectroscopy (EIS) has emerged as a proven effective method for the non-destructive assessment of root morphological parameters (Cao et al., 2010; Cseresnyés et al., 2013). Notably, *in situ* EIS enables the rapid acquisition of root-related parameters without inflicting damage on the root system (Liu et al., 2021), addressing a critical gap in non-invasive seedling evaluation. Concurrently, the quality classification boundaries of *P. orientalis* seedlings are inherently ambiguous. Fuzzy clustering algorithms overcome the “either-or” constraint of traditional hard clustering; rooted in fuzzy set theory, they quantify the uncertainty of an object’s membership across multiple classes, delivering robust clustering results for data approximating a normal distribution (Chen et al., 2020).

Prior studies have validated the feasibility of EIS-based classification for plant-related applications. For instance, Repo et al. utilized the class-featuring information compression (CLAFIC) algorithm to non-destructively classify roots with varying degrees of frost damage using EIS measurement data (Repo et al., 2016). Li et al. measured seven impedance parameters and three physicochemical indices of Fuji apples using an EIS tester, employing support vector machine (SVM) and artificial neural network (ANN) as classifiers to develop a non-destructive detection method for apple moldy core (Li et al., 2013). Cai et al. quantified 126 bioimpedance characteristic values at nine frequency points for apples of varying freshness grades using an LCR meter, employing a sparse principal component analysis-linear classifier (SPCA-LDC) model for classification (Cai et al., 2013). Collectively, these studies demonstrate that electrical impedance spectroscopy (EIS) data can effectively reflect the cellular status of plant tissues, thereby confirming the viability of EIS-based classification. However, existing algorithms demonstrate significant limitations: the CLAFIC algorithm lacks adaptive learning and adjustment capabilities and is sensitive to subspace dimensionality; support vector machine (SVM) and artificial neural network (ANN) algorithms are strongly dependent on parameter selection, requiring intricate tuning procedures; and the SPCA-LDC algorithm is excessively sensitive to outliers and exhibits suboptimal performance with non-linear data. Furthermore, while the fuzzy C-means (FCM) clustering algorithm has demonstrated efficacy for classification tasks with ambiguous boundaries (Aarthi and Sivakumar, 2020; Cardone et al., 2021; Kumar and Ziauddin, 2021), the traditional FCM algorithm requires *a priori* specification of the number of clusters, is sensitive to initial cluster center selection, and tends to converge to local optima—factors that constrain its classification accuracy. Therefore, targeted optimization of the FCM algorithm is imperative to enhance its applicability for grading *P. orientalis* seedlings.

**Figure 1:**
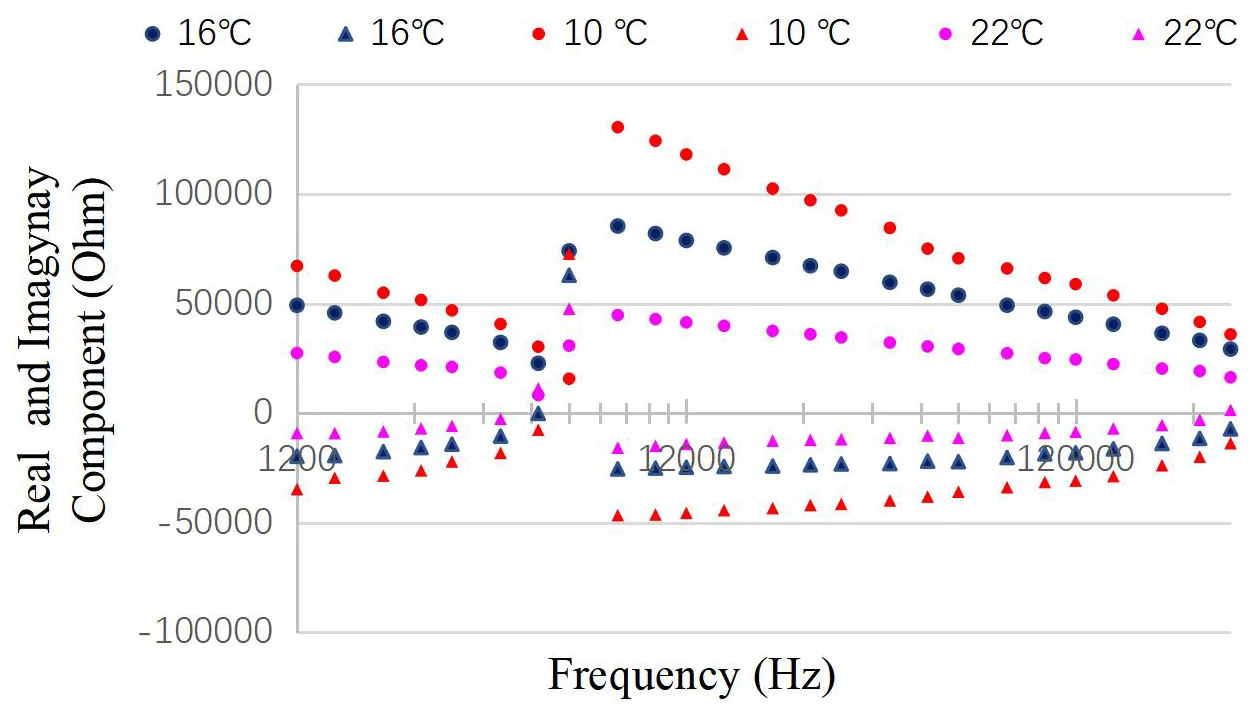
Value of (decreasing) real and (increasing) imaginary component of root EIS of *Platycladus orientalis* under low temperature stress.

In this context, the present study was undertaken. To establish a scientific grading system for *P. orientalis* seedlings, it is first necessary to identify which electrical impedance spectroscopy (EIS) parameters are most sensitive to root quality. Therefore, we manually classified *P. orientalis* seedlings from North China into three grades (Grade I, II, and III) based on conventional morphological criteria and subjected them to different controlled stress conditions. This stress experiment served to examine how varying stress levels-which mimic natural quality degradation-affect the EIS characteristics of roots, thereby enabling the selection of key EIS parameters that effectively discriminate root quality. The results confirmed the sensitivity of EIS to stress (**Figure 1**), allowing us to determine suitable measurement frequencies and candidate parameters for subsequent modeling. Following this parameter screening, we utilized *in situ* EIS technology for non-destructive detection of roots from ungraded *P. orientalis* seedlings. By integrating the selected EIS parameters with other morphological traits, an enhanced fuzzy C-means (FCM) clustering algorithm was implemented to determine the optimal grading thresholds for *P. orientalis* seedlings.

## 2 Materials and methods

### 2.1 Plant material

The plant material employed in this study was *P. orientalis Franco*. All *P. orientalis* individuals were acquired from a commercial nursery located in Mancheng District, Baoding City, Hebei Province, China, which specializes in the cultivation and sale of common horticultural species. As the samples were obtained from a legally operated commercial nursery (i.e., not collected from wild populations) and *P. orientalis* is not listed in the Appendices of the Convention on International Trade in Endangered Species of Wild Fauna and Flora (CITES) (https://www.cites.org/) nor protected under any national or local biodiversity conservation legislation in the People’s Republic of China, no special permits or licenses were required for the procurement of this plant material.

Formal taxonomic identification of the *P. orientalis* samples was performed by Professor Bao Di from the College of Horticulture, Hebei Agricultural University, China, based on morphological characteristics, consistent with the taxonomic description provided in Flora of China (Vol. 4, pp. 61-62).

All experimental procedures involving *P. orientalis*, (including the handling and cultivation of nursery-procured plants, complied with the institutional guidelines established by the Institutional Ethics Committee for Plant Research of Hebei Agricultural University and adhered to relevant national regulations governing agricultural and horticultural research in China.

The experimental nursery is situated in Mancheng District, Baoding, Hebei Province, China (115.28°N, 38.86°E), characterized by a warm temperate semi-humid to semi-arid continental monsoon climate with four distinct seasons, ample sunlight, and synchronous rain and heat periods. The mean annual temperature is 12.9°C, with annual sunshine duration of 2412.7 hours, a frost-free period of 190 days, and average annual precipitation of 546.5 mm.

**Figure 2:**
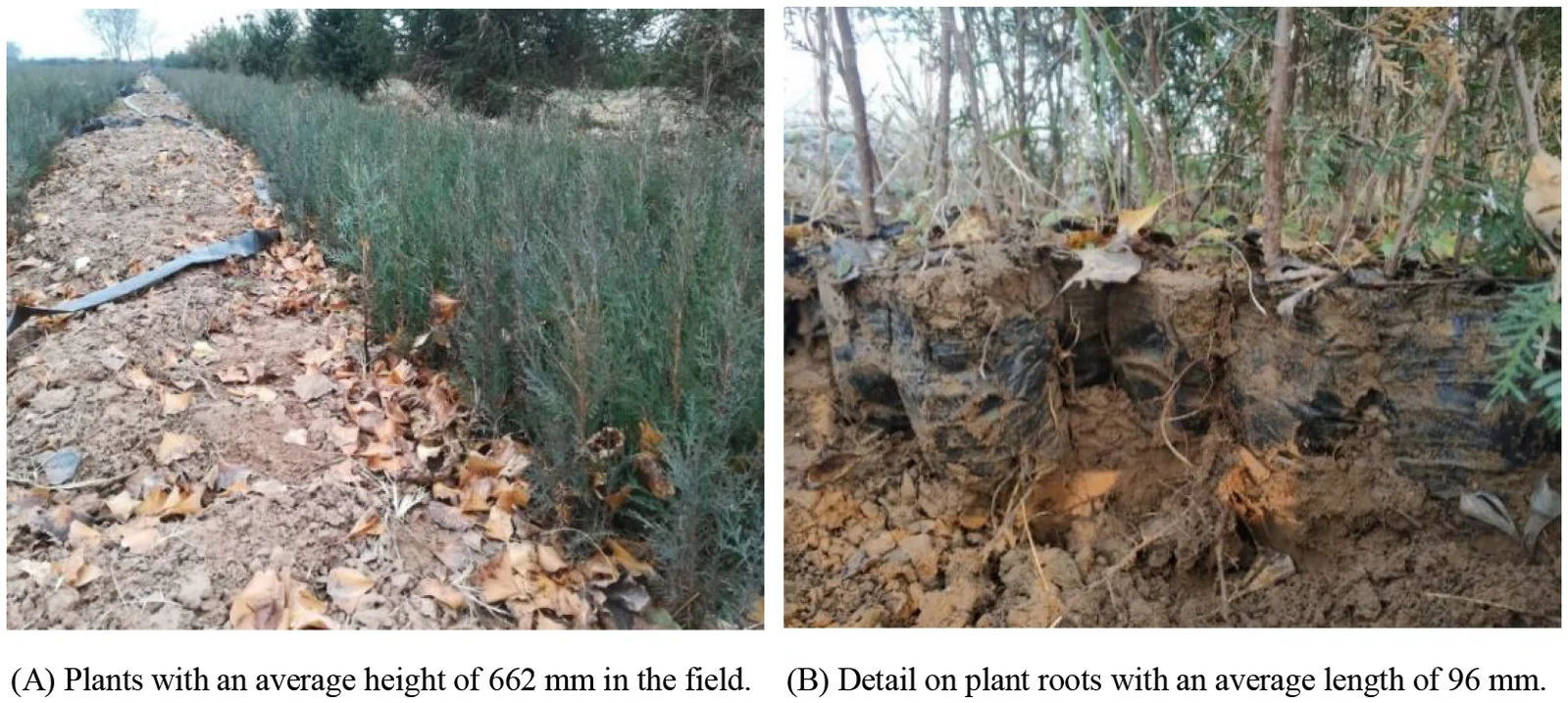
Average height **(A)** and root details **(B)** of 8-months Platycladus orientalis seedlings in the Mancheng Nursery before transplantation for the experiment.

As illustrated in **Figure 2**, eight-month-old *P. orientalis seedlings* were utilized as experimental material. The cultivation substrate consisted of cinnamon soil (pH 8.0) containing 3% organic matter, with seedlings transplanted into nutrient pots 13 cm in diameter.

### 2.2 Experimental treatment and sample preparation

#### 2.2.1 Sample cultivation and selection

**Figure 3:**
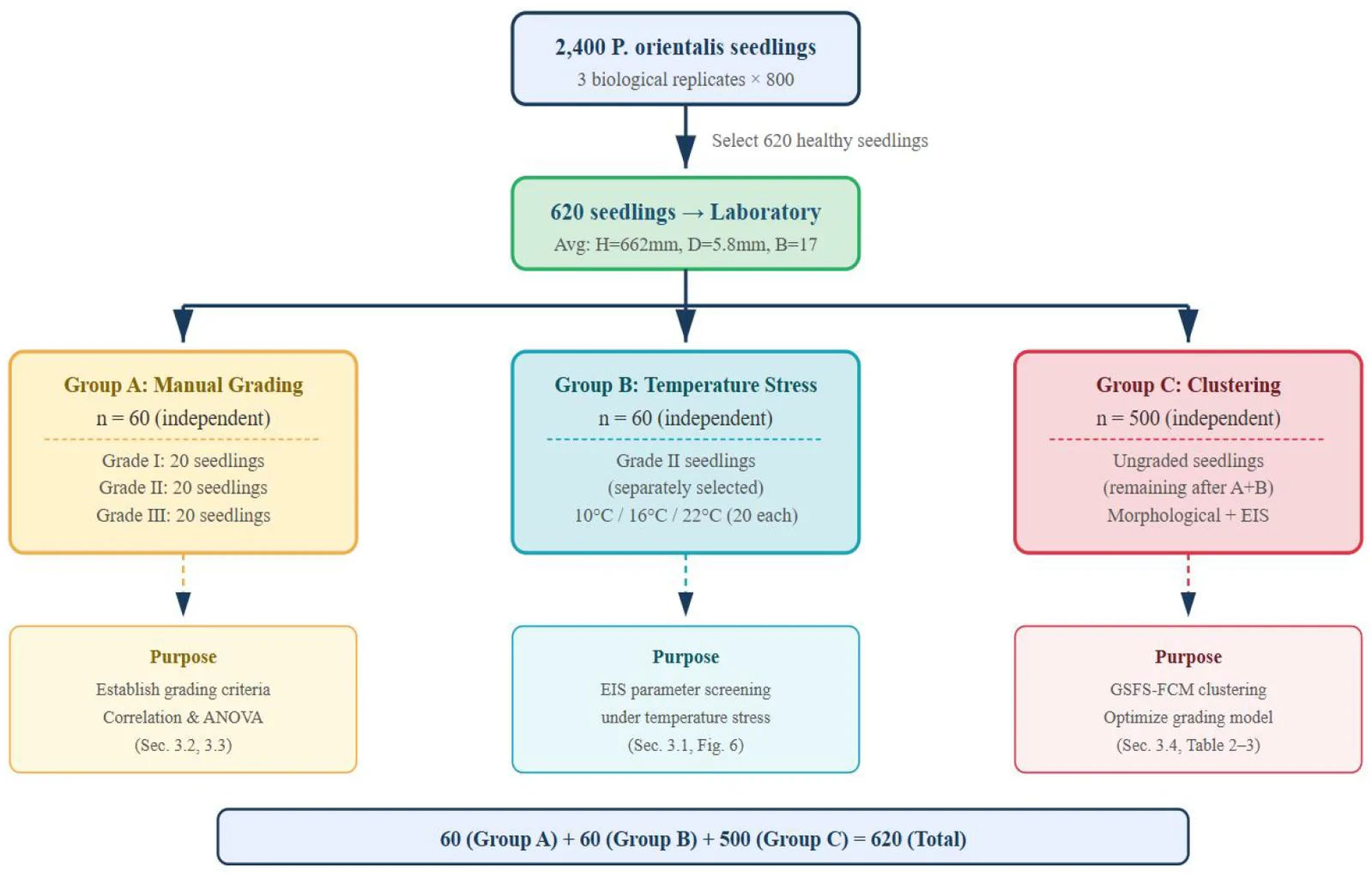
Sample flow diagram.

A total of 2400 P*. orientalis* seedlings were cultivated at the Mancheng Nursery (Baoding, Hebei Province, China). The experiment consisted of three biological replicates, each containing 800 uniformly grown seedlings. On the sampling date, when ambient temperatures ranged from –2°C to 5°C, 620 healthy seedlings were selected and transported to the laboratory of Hebei Agricultural University for subsequent measurements. The average height, stem diameter, and branch number of the 620 seedlings were 662 mm, 5.8 mm, and 17, respectively. These 620 seedlings were allocated to three completely independent experimental groups with no overlap of individual seedlings: (i) 60 seedlings for manual grading (Section 2.2.2), (ii) 60 Grade II seedlings for the temperature stress experiment, and (iii) 500 ungraded seedlings for comprehensive parameter assessment and clustering. The sample-flow diagram is illustrated in **Figure 3**.

#### 2.2.2 Manual grading of seedlings

From the 620 transported seedlings, 60 seedlings were randomly selected (hereafter referred to as Group A) and manually categorized into three distinct grades based on established morphological and electrical impedance spectroscopy (EIS) measurement protocols. The grading criteria were defined as follows: Grade I: stem diameter > 6.5 mm, height > 700 mm, branch number > 20; Grade II: stem diameter 4.5–6.5 mm, height 500–700 mm, branch number 15–20; Grade III: stem diameter < 4.5 mm, height < 500 mm, branch number < 15. Each grade comprised 20 seedlings, which were subsequently subdivided into five groups of four seedlings each. These groups served as biological subsamples for within-grade variability assessment, with four seedlings per subsample to ensure the stability and representativeness of each measurement. These 60 seedlings (Group A) were used exclusively for establishing grading criteria and conducting correlation and ANOVA analyses; they were not used in any subsequent experiments.

#### 2.2.3 Artificial climate chamber treatment

From the remaining 560 seedlings (after removal of the 60 seedlings allocated to Group A), 60 seedlings meeting the Grade II morphological criteria (stem diameter 4.5–6.5 mm, height 500–700 mm, branch number 15–20) were selected as a separate, independent batch (hereafter referred to as Group B). It should be emphasized that these 60 Grade II seedlings (Group B) are distinct from the 20 Grade II seedlings within Group A (Section 2.2.2); no individual seedling was shared between the two groups.

A total of 60 Grade II seedlings (Group B; see Section 2.2.1 for sample allocation) selected as homogeneous experimental material to minimize initial phenotypic variability, were transferred into three artificial climate chambers (MGC-350HP, Shanghai Yiheng Scientific Instrument Co., Ltd., Shanghai, China) located at Hebei Agricultural University (115.44°N, 38.82°E). One chamber was assigned to each temperature treatment (10°C, 16°C, and 22°C), with 20 seedlings per chamber. All three chambers were of the identical model and were calibrated prior to the experiment to ensure uniform performance in lighting (12h photoperiod, 400 μmol·m^-^²·s^-^¹ photosynthetically active radiation), relative humidity (60 ± 5%) (Yao et al., 2025).

The three temperatures were selected based on the known thermal biology of *P. orientalis*. The optimal growth temperature range for this species is 15–25°C (Li et al., 2016), with an optimal annual mean temperature of approximately 14°C (Li et al., 2016). Growth initiation occurs at approximately 10°C, and metabolic activity ceases below this threshold (Li et al., 2016). Accordingly, 22°C was selected as a near-optimal reference temperature (within the optimal growth range); 16°C as a suboptimal temperature (near the upper boundary of the optimal annual mean range, representing mild temperature limitation); and 10°C as a cold stress temperature (at the growth cessation threshold, above the freezing point to avoid irreversible freeze injury). This gradient thus spans a spectrum from cold stress to near-optimal conditions, enabling the identification of EIS parameters sensitive to temperature-mediated physiological changes. Temperatures below 0°C were deliberately excluded to prevent freeze-thaw tissue damage, which would confound reversible stress responses with necrosis. This approach is consistent with previous EIS studies on conifer seedlings (Li et al., 2016).

The seedlings were subjected to three temperature treatments: 10°C, 16°C, and 22°C, with 20 seedlings assigned to each temperature regime (further subdivided into 5 groups of 4 seedlings per group). It should be noted that these five groups within each chamber served as biological subsamples to characterize within-treatment variability among individual seedlings, rather than as independent treatment replicates. The chamber (n 1 per temperature) is the experimental unit for the temperature treatment; consequently, the temperature effect is confounded with the chamber effect, and the statistical analyses on these data should be interpreted as descriptive characterizations of EIS parameter sensitivity rather than as rigorous inferential tests of between-treatment differences. The environmental conditions maintained in the artificial climate chambers during the 30-day temperature treatment experiment are shown in **Table 1**.

**Table 1:** Environmental conditions maintained in the artificial climate chambers during the 30-day temperature treatment experiment.

Parameter	Set value	Notes
Temperature treatment	10°C/16°C/22°C	Constant (no day/night differential); one chamber per treatment
Photoperiod	12 h light/12 h dark	Simulating late-winter/early-spring natural photoperiod
Light intensity (PAR)	400 μmol·m^-^²·s^-^¹	Standard for conifer seedling cultivation
Relative humidity	60 ± 5%	Uniform across all chambers
VSWC (soil moisture)	32% (target)	Monitored daily at 18:00; irrigated to maintain target
Pot arrangement	4 × 5 grid; 2 cm spacing	Randomized initial positions; clockwise rotation every 3 days
Experimental duration	30 days	From treatment initiation to EIS measurement

Pot positions within each chamber were randomized at the start of the experiment. To minimize positional effects arising from potential microenvironmental gradients (e.g., light intensity, airflow) within each chamber, pots were rotated every 3 days following a predetermined clockwise rotation scheme.

Volumetric soil water content (VSWC) was monitored daily at 18:00 using a soil moisture meter (TDR100, Spectrum Technologies, Inc., Plainfield, IL, USA). Following each measurement, supplemental irrigation was applied to maintain the soil water content at a target level of 32% throughout the experimental period.

#### 2.2.4 Parameter measurement arrangement

Following a 30-day cultivation period in artificial climate chambers, the root electrical impedance spectroscopy (EIS) parameters of the Grade II seedlings (Group B) were quantified. After the removal of the 60 seedlings allocated to Group A (Section 2.2.2) and the 60 seedlings allocated to Group B (Section 2.2.3), the remaining 500 seedlings (hereafter referred to as Group C; 620 − 60 − 60  500) ungraded seedlings underwent comprehensive assessments of both morphological and EIS parameters, constituting the core dataset for optimizing the seedling grading model. Group C seedlings were not subjected to any prior grading or temperature treatment, ensuring an unbiased dataset for clustering analysis.

#### 2.2.5 Field validation design

##### 2.2.5.1 Source and independence of validation seedlings

To evaluate the field performance of the five-grade classification system, an independent set of 450 P*. orientalis* seedlings was selected from the remaining nursery stock (1, 780 seedlings not transported to the laboratory; see Section 2.2.1) at the Mancheng Nursery (115.28°N, 38.86°E). These validation seedlings were not part of the 500 seedlings used for GSFS-FCM model development (Group C), ensuring true external validation. An additional 270 seedlings were allocated to the traditional three-grade system as a control comparison. The total validation cohort comprised 720 seedlings.

##### 2.2.5.2 Grading and allocation

All 450 validation seedlings were measured for the five feature variables (stem diameter, plant height, branch number, θ @ 4 kHz, tan δ @ 400 kHz) and assigned to Grades 1–5 using the GSFS-FCM clustering centers established from the model development set (**Table 2**). The 270 control seedlings were graded using the traditional morphological criteria (Grade I: stem diameter > 6.5 mm; Grade II: 4.5–6.5 mm; Grade III: < 4.5 mm).

**Table 2:** Eigenvalues and variance explanation of all 13 principal components.

PC	Representative variable (highest |loading|)	Eigenvalue	Variance (%)	Cumulative (%)	Decision (Kaiser: eig > 1)
PC1	Stem diameter	4.748	36.519	36.519	Retain
PC2	Plant height	2.335	17.965	54.484	Retain
PC3	Branch number	1.607	12.361	66.845	Retain
PC4	4 kHz phase angle	1.283	9.873	76.718	Retain
PC5	400 kHz tan δ	1.007	7.744	84.461	Retain
PC6	400 kHz phase angle	0.725	5.576	90.037	Discard
PC7	Trunk height	0.440	3.385	93.422	Discard
PC8	1st branch diameter	0.346	2.664	96.087	Discard
PC9	1st branch length	0.222	1.704	97.791	Discard
PC10	Top 20 cm fresh weight	0.149	1.149	98.940	Discard
PC11	Top 20 cm removed fresh weight	0.091	0.697	99.637	Discard
PC12	2nd branch diameter	0.046	0.352	99.989	Discard
PC13	2nd branch length	0.001	0.011	100.000	Discard

Validation of 5-PC selection — two converging criteria:.

I Kaiser criterion: exactly 5 PCs (PC1–PC5) have eigenvalues > 1; the gap between PC5 (1.007) and PC6 (0.725) is 0.282, clearly separating retained from discarded components.

II Cumulative variance threshold (≥ 80%): the 5 retained PCs explain 84.461% of total variance, exceeding the recommended threshold.

##### 2.2.5.3 Field planting and experimental design

The field validation experiment was conducted at the experimental field of Hebei Agricultural University (38°51’25” N, 115°29’13”E). Transplanting was carried out on March 15, 2023, approximately two weeks after the initial sampling date (when the ambient temperature was –2°C to 5°C; Section 2.2.1). At this time, daily mean temperatures had risen to 3–8°C, suitable for transplanting. The soil at the experimental site was cinnamon soil (pH 8.0, 3% organic matter), consistent with the nursery substrate used for seedling cultivation. The validation period extended for 180 days, from March 15 to September 10, 2023, covering the complete spring-to-autumn growing season.

The experiment was arranged in a randomized complete block design with three blocks. Within each block, plots (2 m×2 m) were randomly assigned to the five grades of the new classification system and the three grades of the traditional system. The randomization was independently generated for each block using a random number table. Each plot contained 30 seedlings planted at 30cm×30cm spacing. A 50 cm buffer zone was maintained between adjacent plots to minimize edge effects.

For the five-grade validation cohort (n 450), 90 seedlings were assigned to each grade (3 plots × 30 seedlings per plot). For the traditional three-grade control (n 270), 90 seedlings were assigned to each grade (3 plots×30 seedlings per plot). The seedling allocation and per-plot survival data are provided in **Supplementary Table 1**.

All seedlings received uniform management practices throughout the validation period, including irrigation (supplemental watering during dry periods to maintain soil moisture at 60–70% field capacity), fertilization (compound fertilizer N:P:K 15:15:15 at 20 g per seedling applied at transplanting and again at 90 days), and weed control (manual weeding every 30 days). No phytosanitary treatments were applied during the validation period.

##### 2.2.5.4 Data collection

Survival status and growth parameters (plant height increment, stem diameter increment, and branch number increment) were recorded at the end of the 180-day validation period. The number of surviving seedlings per plot is provided in **Supplementary Table 1**. The experimental unit for all statistical analyses was the plot (n 3 per grade), not the individual seedling.

### 2.3 Measured parameters

#### 2.3.1 Aboveground morphological parameter

Based on the Chinese national seedling grading standards and relevant literature, the following shoot morphological parameters were selected for *P. orientalis* (Guo, 2008; Cao et al., 2010; Repo et al., 2016; Di et al., 2019; Liu et al., 2021): fresh weight (g), stem diameter (mm), plant height (mm), number of branches, trunk height (mm), thickness of the first branch (mm), length of the first branch (mm), thickness of the second branch (mm), length of the second branch (mm), fresh weight of the top 20 cm (g), dry weight of the top 20 cm (g), fresh weight after removing the top 20 cm (g), and dry weight after removing the top 20 cm (g).

#### 2.3.2 Root morphological parameter

Based on the findings from Repo (Repo et al., 2005), parameters including primary root length, primary root thickness, root length, root volume, root average diameter, root surface area, and the number of lateral roots were selected for quantification. Root length, root volume, root average diameter, and root surface area were measured utilizing a root scanner (WinRhizo, Regent Instruments, Quebec, Canada; WinRHIZO 2016 software). Subsequently, the roots were oven-dried at 80°C for 48 hours to determine the dry weight (Ozier-Lafontaine and Bajazet, 2005).

#### 2.3.3 Root EIS parameters

**Figures 4:**
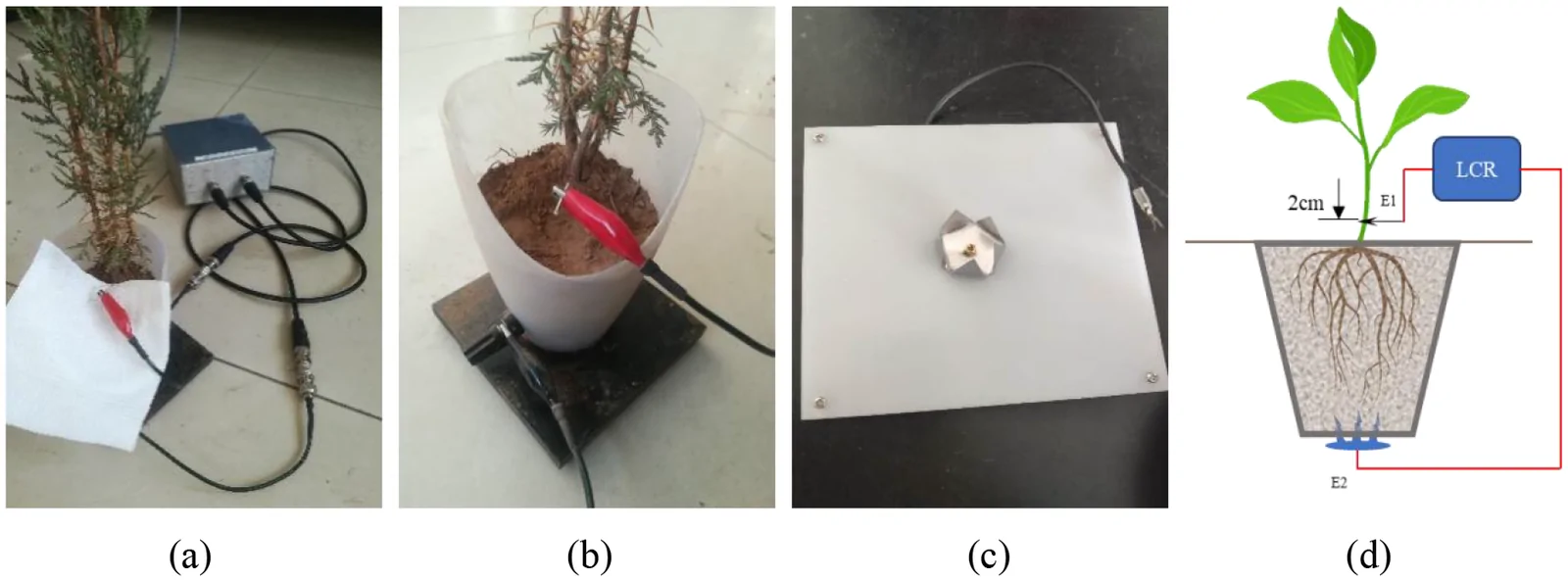
Seedlings in soil containers at the laboratory of Hebei Agricultural University for the electrical impedance spectroscopy (EIS) experiment. Schematic diagram of EIS measurements of *Platycladus orientalis*. **(a)** Actual scenario **(b)** electrode connection **(c)** base electrode **(d)** measuring principle.

The EIS-100 system (Simitec Oy/Simitec Ltd, Finland) was employed to measure the electrical impedance parameters of seedling roots. This system is capable of acquiring impedance spectra with up to 512 frequency points across a frequency range of 1000 Hz to 1 MHz, with an accuracy of 0.1 Hz. Before measurements, the EIS-100 system was calibrated according to the manufacturer’s instructions. For the measurements, container seedlings, together with the soil, were inserted into the stainless steel electrode base (Ozier-Lafontaine and Bajazet, 2005; Repo et al., 2005; Guo, 2008; Repo et al., 2014; Di et al., 2019). The root systems were measured together with the surrounding substrate, and the original root–soil configuration was maintained during EIS acquisition. A stainless steel needle electrode (diameter 0.3 mm, Kangsheng Europe GmbH, Wiesbaden, Germany) was inserted vertically into the substrate at a fixed position 2 cm above the root–soil junction (Ozier-Lafontaine and Bajazet, 2005), as illustrated in **Figure 4**. The insertion depth and position were kept consistent among all seedlings to maintain comparable electrode contact conditions and measurement geometry. After electrode placement, the system was allowed to stabilize for 10 min before impedance acquisition. The measurement circuit comprised a stem section, the stem-soil interface, the root, the root-soil interface, the soil, and the base electrode. The EIS-100 system conducted electrical impedance measurements using a voltage excitation of 100 mV over a frequency spectrum from 1200 Hz to 1 MHz. For each seedling, 3 impedance spectra were collected as technical replicates, and the averaged spectrum was used for subsequent analysis. Measurements were conducted at approximately 25°C.

The selection of EIS parameters (e.g., phase angle, tan δ, impedance magnitude at specific frequencies) was based on previous studies that established equivalent circuit models (e.g., including Warburg and constant phase elements) to interpret their physiological relevance to root membrane integrity, vacuole size, and overall vitality (Repo et al., 2005; Cao et al., 2010; Di et al., 2019; Liu et al., 2024). Thus, these parameters are employed as correlated proxies for root quality in the present grading model noting that direct physiological validation (e.g., membrane integrity assays, root respiration) was not performed in this study, and no new equivalent circuit model was constructed.

### 2.4 Data analysis

Data preprocessing, encompassing the cleaning of morphological parameters and electrochemical impedance spectroscopy (EIS) parameter values derived from *P. orientalis*, was performed to facilitate subsequent data analysis, data mining, and clustering algorithm applications. All 500 seedlings in Group C were retained for clustering analysis ( ${\text{n}}_{1}$ 65, ${\text{n}}_{2}$ 81, ${\text{n}}_{3}$ 105, ${\text{n}}_{4}$ 81, ${\text{n}}_{5}$ 96, ${\text{n}}_{6}$ 72 after cluster assignment). Statistical analyses were conducted using IBM SPSS Statistics version 20. Correlation analysis was employed to investigate the relationship between root EIS values and root morphological parameters in *P. orientalis*, with a significance level set at α 0.05. Given the large number of pairwise correlations computed across multiple EIS parameters, root traits, and frequency points, the Benjamini-Hochberg false discovery rate (BH-FDR) correction (Benjamini and Hochberg, 1995) was applied to control the proportion of false positives among the declared significant correlations. The FDR was controlled at Q  0.05. Bonferroni correction was also reported as a conservative reference. Correlations surviving both correction methods were considered robust; frequencies selected based on correlations not surviving correction were further validated using an independent dataset.

One-way analysis of variance (ANOVA) followed by Least Significant Difference (LSD) *post-hoc* tests was utilized to assess the significance of differences among data corresponding to varying soil water content levels, at a significance level of α 0.05. Given that each temperature treatment was conducted in a single climate chamber (n 1 chamber per treatment), the five subgroups within each chamber represent biological subsamples rather than independent chamber replicates. The ANOVA results should therefore be interpreted as descriptive comparisons of EIS parameter sensitivity across temperature regimes, not as rigorous inferential tests of temperature effects per se. The primary purpose of this analysis was to screen candidate EIS parameters for subsequent use in the clustering-based grading model.

Prior to principal component analysis (PCA), all variables were Z-score standardized (mean 0, SD  1) given their heterogeneous measurement units. A total of 16 variables were measured (13 morphological and 3 EIS-derived). Three morphological variables — total fresh weight, dry weight of the top 20 cm section, and dry weight after removal of the top 20 cm section — were excluded prior to PCA due to perfect or near-perfect multicollinearity (total fresh weight is an exact linear combination of its two components; the two dry-weight variables each correlate with their corresponding fresh-weight variable at r > 0.95). The remaining 13 variables (10 morphological + 3 EIS) were subjected to PCA.PCA was performed on the correlation matrix using IBM SPSS Statistics 20.0. The number of principal components (PCs) to retain was determined by the Kaiser criterion (eigenvalue > 1) combined with a cumulative variance threshold of ≥ 80% (Liu, 2016). Variable selection from the PCA results followed the Jolliffe method (Jolliffe, 1972) for each retained PC, the original variable with the highest absolute rotated loading (|λ| ≥ 0.70) was identified as the representative variable; variables requiring destructive measurement were replaced by the next-highest-loading non-destructive alternative on the same component; and all selected variables were cross-validated against ANOVA and correlation analysis results. This procedure ensured an objective, reproducible link between PCA dimensionality reduction and final variable selection.

Summary, the EIS variable- and frequency-selection procedure consisted of four sequential steps: (i) Pearson correlation analysis between each EIS parameter (θ, tan δ, impedance magnitude) at each measurement frequency and seven root morphological traits, with significance set at p < 0.05 (two-tailed); (ii) application of five selection criteria (statistical significance, trait coverage breadth, frequency-band complementarity, non-redundancy, and grade discriminative power—see Section 3.3.1 for details) to identify candidate parameters; (iii) one-way ANOVA with LSD *post-hoc* tests to confirm that selected parameters significantly discriminate among manual seedling grades (P < 0.05); and (iv) PCA to verify that selected parameters contribute unique variance to the principal components.

### 2.5 Algorithm description and integration for seedling grading

The primary objective of this research was to establish an automated, precise, and non-destructive grading system for *P. orientalis* seedlings through the integration of electrical impedance spectroscopy (EIS) parameters with essential morphological traits. Owing to the inherent fuzziness in seedling quality boundaries and the limitations of conventional clustering methods in processing multi-dimensional seedling data, an enhanced Fuzzy C-Means (FCM) clustering framework was proposed to facilitate seedling quality grading.

#### 2.5.1 Fuzzy C-means clustering algorithm optimized by genetic sparrow family search

The traditional Fuzzy C-Means (FCM) algorithm requires manual specification of cluster centers and a predetermined number of clusters (Liu and Xu, 2018). The Sparrow Search Algorithm (SSA) exhibits robust local search capability and rapid convergence speed. However, each sparrow converges to the current optimal solution by directly jumping to its vicinity, which predisposes the algorithm to local optima and compromises its global search performance (Xue and Shen, 2020). Furthermore, the quality of the initial population influences the subsequent optimization efficiency, and the random initialization method employed in the sparrow optimization algorithm fails to ensure uniformity and diversity within the initial population. Consequently, this paper proposes a fuzzy C-means clustering algorithm based on genetic sparrow family search optimization (GSFS-FCM).

**Figure 5:**
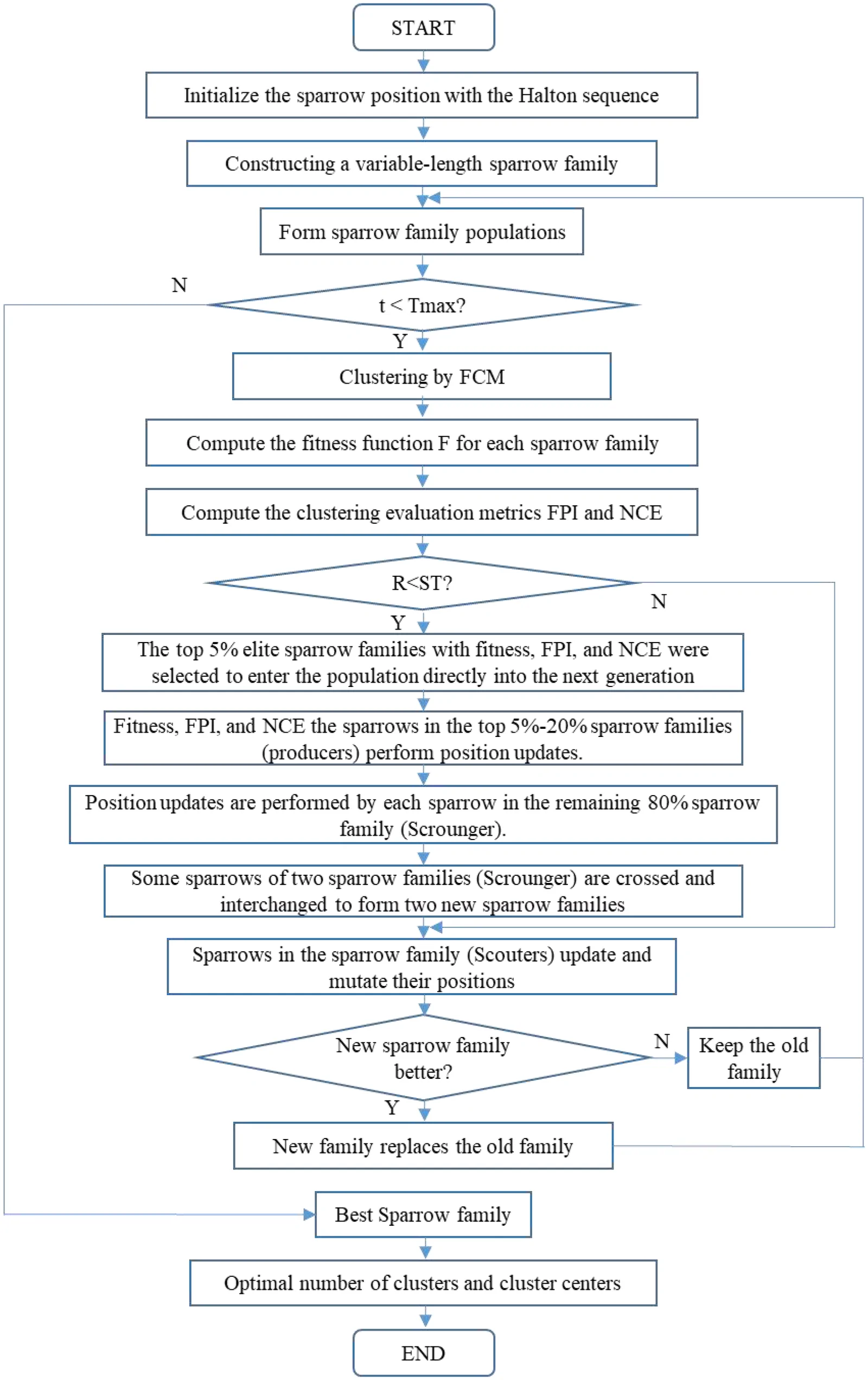
GSFS-FCM algorithm model.

By integrating genetic algorithm and sparrow algorithm at the preliminary stage, the GSFS-FCM algorithm combines the strong global search ability of genetic algorithm with the fast local search speed of sparrow algorithm. Additionally, it addresses the limitations of the FCM clustering algorithm, namely its inability to autonomously determine the number of clusters and its sensitivity to initial cluster centers, through a variable-length sparrow family coding method. The sparrow algorithm is further refined via multiple strategic optimizations. The specific algorithmic model is illustrated in **Figure 5**.

In the integration of the genetic algorithm and the Sparrow algorithm, it is imperative to account for critical elements during the implementation process, including population initialization, coding schemes, fitness functions, as well as genetic crossover and mutation operators, all of which substantially influence algorithmic efficiency. Concurrently, enhancements are applied to the algorithm in the following aspects:

##### 2.5.1.1 Halton sequence initialization

**Figure 6:**
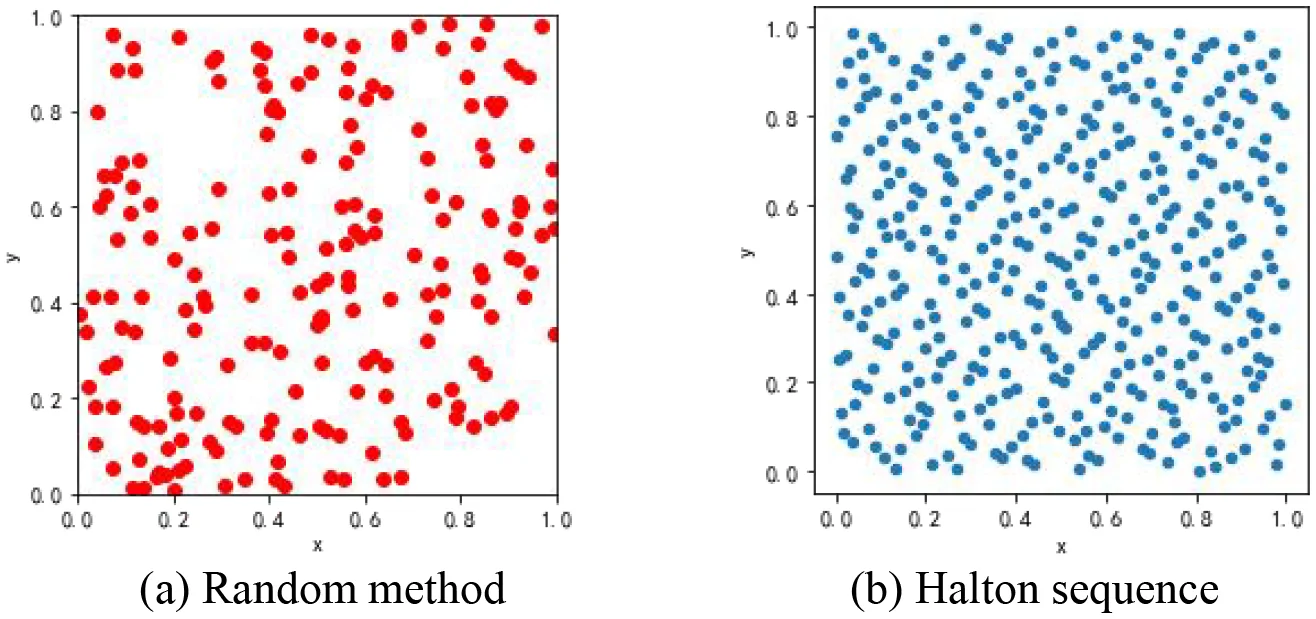
Distribution of individuals generated by Random method **(a)** and Halton sequence **(b)**.

The conventional sparrow search algorithm employs a random population initialization strategy, which frequently leads to an uneven distribution of individuals within the search space. This often results in initial positions clustering near local optima, thereby predisposing the algorithm to premature convergence and local optimum entrapment. In contrast, the Halton sequence is a low-discrepancy sequence capable of generating a uniformly distributed set of points across the solution domain (Zhang et al., 2017; Wang et al., 2020). A comparative visualization of two-dimensional populations initialized via random sampling versus the Halton sequence is presented in **Figure 6**.

A comparison between **Figures 6a, b** reveals that individuals generated via the Halton sequence exhibit a more uniform distribution across the two-dimensional space (Zhang et al., 2017; Aarthi and Sivakumar, 2020). Consequently, this study employs the Halton sequence for population initialization, thereby ensuring population diversity, enhancing the global search capability, improving algorithmic stability, and effectively mitigating the risk of convergence to local optima.

##### 2.5.1.2 Sparrow family variable length coding scheme

In addressing clustering problems, the optimal number of clustering centers is inherently difficult to determine and is typically established based on empirical settings. However, reliance on such empirical determinations may introduce bias into the clustering outcomes. Therefore, this study proposes a method in which multiple sparrows are randomly organized into sparrow families. Each sparrow family employs a variable-length genetic coding scheme to dynamically determine the number of clustering centers. The permitted range of cluster numbers is c ∈ (2, 10), where $c_{min}$ 2 ensures meaningful partitioning and $c_{max}$ 10 is set based on the sample size (n 500) to ensure adequate cluster membership. Each sparrow family’s chromosome length equals its randomly assigned cluster count sampled from (2, 10). The corresponding chromosomes of the sparrow families are implemented using this variable-length coding strategy.

##### 2.5.1.3 Weighted probability producer location update mechanism

For producer sparrow families, defined as those ranked within the top 5% to 20% based on fitness, the position of each member is updated according to the enhanced formulation (Liang et al., 2021). Concurrently, to strengthen both global and local exploration capabilities of the algorithm, a generation-dependent weight ω is introduced (Liang et al., 2021).

(1) $$\omega =1-\frac{\left(exp(\frac{t}{t_{max}})-1)*\ln(4\right)}{e}$$

**Figures 7:**
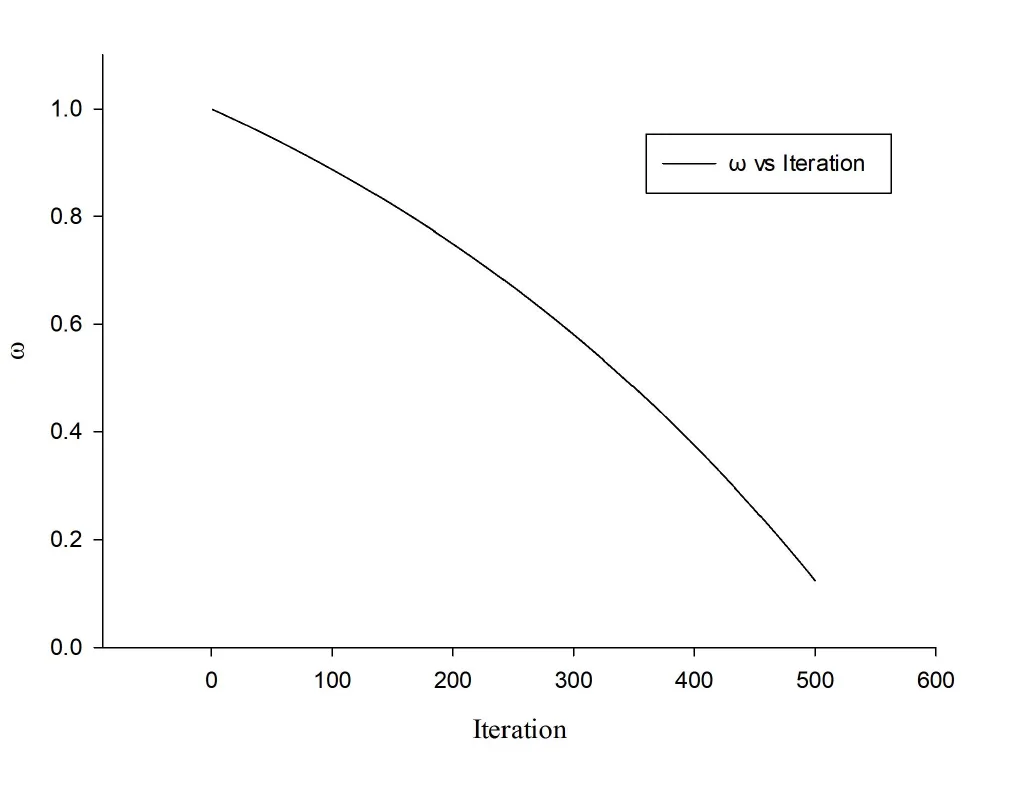
Curve for the values of inertia weight (ω) against the number of iterations. (between 0 and *t_max_*).

In this context, *t* denotes the current iteration number, and *t_max_* represents the maximum number of iterations. The inertia weight ω adaptively decreases with increasing iteration count, as illustrated in **Figure 7**. The incorporation of the inertia weight facilitates adaptive control over its value range.

Furthermore, an out-of-bounds handling mechanism is implemented. Specifically, if a sparrow member’s position exceeds the predefined boundaries, a random position adjacent to the current optimal position is assigned, such that the new position is $X_{new}$:

(2) $$X_{new}=X_{best}+|X_{best}-X_{good}|\cdot rand(1)$$

Where $X_{best}$ is the global optimal position and $X_{good}$ is the current optimal position.

#### 2.5.2 Detailed description of key operators and algorithm parameters

##### 2.5.2.1 Mutation operator

A non-uniform mutation operator is applied to the scouters (sparrows aware of danger) with a dynamically decreasing probability $P_{m}$ from 5% to 2% over iterations. For a selected sparrow family member $x_{ij}$ (the j-th coordinate of the  i-th individual), the mutated value is:

(3) $$x_{ij}^{'}=x_{ij}+\Delta (t,\;bound_{j})$$

where $bound_{j}$ is the width of the feasible interval for the j-th dimension. The perturbation magnitude Δ(t,  y) decays with iteration count:

(4) $$\Delta (t,\;\;y)=y\cdot (1-r^{{\left(1-t/t_{max}\right)}^{b}})$$

Here r is a uniformly distributed random number in (0, 1), b 3 is a shape parameter, and $t_{max}$=G=50. This operator allows large exploration in early iterations and fine-tuning in later iterations.

##### 2.5.2.2 Elitism strategy

After evaluating the fitness, the top 5% of sparrow families (elite individuals) are directly copied to the next generation without undergoing crossover or mutation. The GSFS-FCM algorithm employs three independent evaluation indices to assess clustering quality:

Fitness function (F):

(5) $$F=\frac{1}{1+\sum_{i=1}^{Len(x_{i})}\sum_{j=1}^{g}{\left(\mu_{ji}\right)}^{m}∥x_{j}-\omega_{i}{∥}^{2}}$$

where c chromosome length (cluster number), n number of data samples, m fuzzification parameter, ${\omega \;}_{i}$ cluster center, $\mu_{ij}$ membership degree. Lower F indicates better clustering.

Fuzzy Performance Index (FPI):

(6) $$FPI=1-\frac{c}{c-1}[1-\sum_{i=1}^{g}\sum_{j=1}^{c}\mu_{ji}^{2}/g]$$

Ranges in (0, 1); lower values indicate sharper partitions.

(7) $$H=-\sum_{i=1}^{g}\sum_{j=1}^{c}\mu_{ji}\log_{a}(\mu_{ji})/g$$

Classification entropy; ranges from 0 to log_a_(c).

Normalized Classification Entropy (NCE):

(8) $$NCE=\frac{H}{1-c/g}$$

Normalized classification entropy; lower values indicate better classification.

The optimal cluster centers and cluster number are determined by the solution that simultaneously minimizes F, FPI, and NCE.

##### 2.5.2.3 Complete parameter table

**Table 3** summarizes all algorithm parameters, their symbols, values or dynamic schedules, and descriptions.

**Table 3:** All parameters of GSFS-FCM.

Parameter	Symbol	Value/schedule	Description
Maximum iterations	G ${\text{t}}_{\text{max}}$	50	Total number of generations
Sparrow population size	${\text{GS}}_{\text{size}}$	55	Number of sparrow individuals
Family population size	${\text{GF}}_{\text{size}}$	55	Number of sparrow families
Crossover probability	${\text{P}}_{\text{c}}$	0.8 (80%)	Probability of crossover between two families
Mutation probability range	${\text{P}}_{\text{m}}$	5% → 2% (linear decrease)	Probability of applying mutation to scouters
Elite proportion	${\text{E}}_{\text{n}}$	5%	Proportion of top individuals preserved via elitism
Producer proportion	PD	20%	Proportion of sparrows acting as discoverers
Danger-aware sparrow proportion	SD	20% → 10% (linear decrease)	Proportion of sparrows acting as scouters
Warning threshold	${\text{R}}_{2}$	0.8	Minimum alarm threshold for scouter activation
Inertia weight formula	ω	Equation 1	Non-linear decreasing weight
Mutation shape parameter	b	3	Controls mutation perturbation decay
Number of elite individuals	–	4	Equivalent to (*E_n_ × GFsize*)

Dynamic parameter update formulas.

$P_{m}(t)=0.05-(0.05-0.02)\cdot t/t_{\max}$, $SD(t)=0.20-(0.20-0.10)\cdot t/t_{max}$.

#### 2.5.3 Integration for seedling grading

The feature vector for clustering was selected through prior correlation analysis and principal component analysis, as it comprehensively captures above-ground morphological vigor and root quality indicators (EIS parameters correlated with root morphological traits)—two core dimensions of seedling quality assessment. Initially, the GSFS optimizer was applied to this feature space to automatically identify a set of robust cluster prototypes. These prototypes were subsequently utilized to initialize the FCM algorithm, which performed the final fuzzy partitioning of the *P. orientalis* seedling dataset. This GSFS-FCM hybrid approach was designed to achieve stable, accurate, and data-driven seedling grading, thereby eliminating the necessity for manual specification of the number of quality grades and enhancing the practical applicability of the system.

The pseudocode for implementing the GSFS-FCM algorithm in grading *P. orientalis* seedlings is presented as follows.

Initialization Procedure:

1. Set the parameters for the GSFS-FCM algorithm as follows:

G: maximum number of iterations for the GSFS-FCM algorithm, set to 50.
GSsize: sparrow population size, set to 55.
GFsize: sparrow family population size, set to 55.
Pc: crossover probability, set to 80%.
Number of elite individuals: 4.
Pm: mutation probability, dynamically adjusted from 5% to 2%.
En: proportion of elite individuals, set to 5%.
PD: proportion of producers (discoverers), set to 20%.
SD: the proportion of sparrows aware of danger, dynamically adjusted from 20% to 10%.
R2: the minimum warning threshold, set to 0.8.

2. Construct the feature weight vector w (w_1_, w_2_,…, w_d_) based on the absolute Pearson correlation coefficient of each feature with primary root diameter (the root parameter most strongly associated with seedling quality):

(9) $$w_{j}=|r_{j}|/\sum_{k=1}^{d}\left|r_{k}\right|\;\text{subject to}\;\sum_{j=1}^{d}w_{j}=1$$

where $r_{j}$ is the correlation coefficient of feature j with primary root diameter (from §3.3.1). The resulting weights for the five selected features are: stem diameter (w_1_ 0.231), plant height (w_2_ 0.185), branch number (w_3_ 0.139), θ @ 4 kHz (w_4_ 0.212), tan δ @ 400 kHz (w_5_ 0.233). These weights are incorporated into the weighted Euclidean distance used by FCM:

(10) $$d_{w}(x_{j},\;v_{k})=\sqrt{\sum_{j=1}^{d}w_{j}}\cdot {\left(x_{ij}-v_{kj}\right)}^{2}$$

3. Define the solution space boundaries for each dimension as: $V_{j}^{min}$ 0, $V_{j}^{max}$ 1 for all *j* ∈{1,…, d}, since all features are normalized to (0, 1) via min-max normalization (see step 4 below). If any coordinate of a sparrow’s position falls outside [0, 1], it is reset using Equation 2; if the reset value still exceeds the boundary, it is clamped to the nearest limit (0 or 1).

4. Normalize all attribute variables using min-max normalization, transforming values to the interval [0, 1]. The normalized value x’ for a variable x is calculated as: ${\text{x}}^{\prime }=(\text{x}-min_{x})/(max_{x}-min_{x})$.

Output: Optimal number of clusters and the optimal cluster center coordinate matrix.

Iterative Search Procedure:

1. Initialize the sparrow population using a Halton sequence.
2. Initialize the sparrow family population utilizing variable-length chromosomes.
3. While the iteration count t has not reached the maximum G:
4. Use the number of members in each initialized sparrow family as the cluster number and the sparrow positions as the initial cluster centers.
5. Execute the Fuzzy C-Means (FCM) algorithm with fuzzification parameter m = 2.0 to perform clustering. The FCM objective function $J_{m}=\sum_{i}\sum_{k}\mu_{ik}^{m}\cdot ∥x_{i}-\omega_{k}{∥}^{2}$ is embedded in the fitness function $F=1/(1+J_{m})$, with membership and center updates given by Equations 9, 10.
6. Compute the fitness function along with the clustering evaluation indices FPI and NCE for each sparrow family (Equations 5–8).
7. Sort all individuals based on their fitness value, FPI, and NCE, and identify the current optimal individual.
8. Select the top 5% of elite sparrow families (determined by fitness, FPI, and NCE) to be preserved directly in the next generation population.
9. For i (top 5% + 1) to 20% of the population:
10. Update the positions of the producers (discoverers).
11. End For
12. For i (top 20% + 1) to 100% of the population:
13. Update the positions of the Scrounger.
14. Perform crossover between two randomly selected sparrow families to generate two new families.
15. End For
16. For i 1: 20% to 10% of the population:
17. Update the positions of the Scouters (sparrows aware of danger).
18. Apply mutation to a subset of the Scouter sparrow families, with probability decreasing from 5% to 2% (Equations 3, 4).
19. End For
20. Form the new sparrow family population for the next generation.
21. If the fitness of a new sparrow family individual is superior to its predecessor, update the corresponding entry in the sparrow family population.
22. The iteration count *tt* + 1.
23. End While
24. Identify the globally optimal sparrow family.
25. Extract the optimal number of clusters and the corresponding optimal cluster centers.

Empty cluster handling: After each FCM iteration, a cluster k is flagged as empty if its membership sum $\sum_{i}\mu_{ik}$< *δ* (*δ*  1.0). The center of an empty cluster is reassigned to the data point farthest from its currently assigned cluster center. If empty clusters persist after three consecutive reinitialization attempts, the cluster count for that sparrow family is reduced by one and the chromosome length is adjusted accordingly.

## 3 Results

### 3.1 Trend of EIS value in roots of *P. orientalis* under temperature stress

As illustrated in **Figure 1**, the EIS of *P. orientalis* roots exhibits marked variations under temperature stress. With the excitation frequency increasing progressively from 1200 Hz, the real *component of the i*mpedance declines gradually, while the imaginary component rises slowly. Within the frequency range of 5000–6000 Hz, both the real and imaginary component of the electrical impedance have an anomalous inflection. Subsequently, as the frequency continues to rise, the real component increases once again, whereas the imaginary component decreases. Up to 300 kHz, the real component demonstrates a slow declining trend, and the imaginary component shows a gradual upward tendency. Beyond 300 kHz, both components exhibit irregular numerical behavior. Throughout the frequency sweep, particularly within the intervals of 1200–4000 Hz and 8000–250 kHz, the real and imaginary components of the root electrical impedance in *P. orientalis* seedlings subjected to low-temperature stress differ significantly. These findings provide empirical support for the application of EIS parameters in characterizing root *quality in P. orientalis* seedlings.

### 3.2 Trend of root characteristic values of different quality *P. orientalis* seedlings

**Figures 8:**
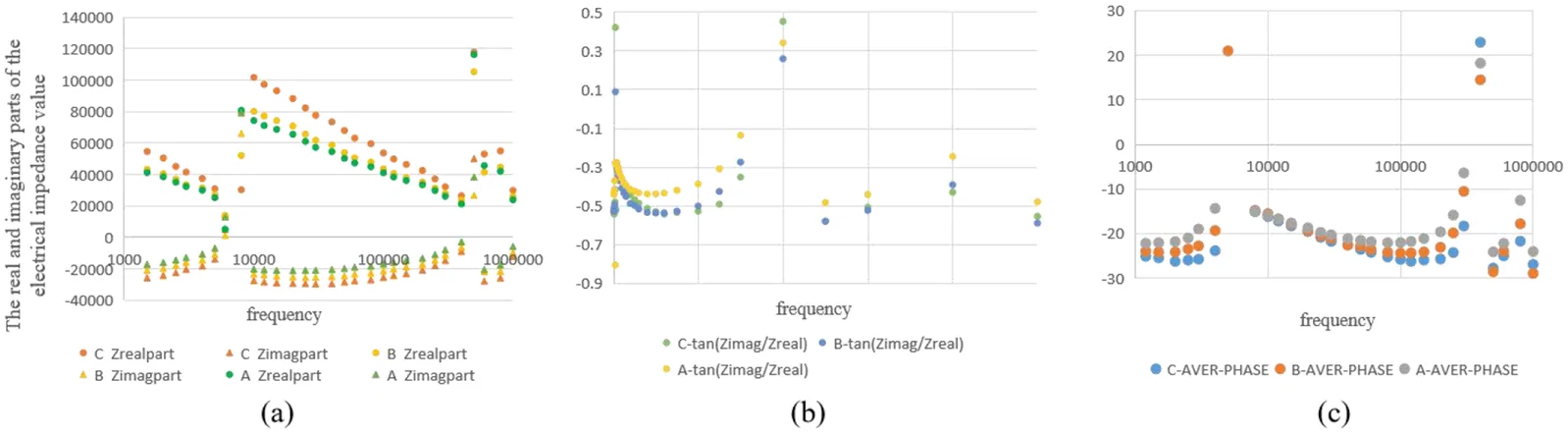
A represents Grade I seedlings, B represents Grade II seedlings, and C represents Grade III seedlings. **(a)** Changes in the real and imaginary component of EIS; **(b)** tan(Zimag/Zreal) change of EIS; **(c)** phase changes of the EIS.

As illustrated in **Figure 8**, *P. orientalis* seedlings cultivated under natural growth conditions were initially classified according to established grading criteria. Subsequently, electrical impedance spectroscopy (EIS) measurements were conducted, wherein A corresponds to Grade I (superior), B to Grade II (medium), and C to Grade III (inferior). It was observed that the variations in the real and imaginary components of root electrical impedance across different grades of *P. orientalis* were consistent with those under stress conditions. The mean electrical impedance tangent (Zimag/Zreal) exhibited a continuous curve over the frequency range of 1200 Hz to 300 kHz, whereas the mean phase angle (θ) was characterized by two distinct continuous curves: from 1200 Hz to 4000 Hz and from 8000 Hz to 300 kHz.

### 3.3 Relationship between root EIS value and root morphological parameters of *P. orientalis*

#### 3.3.1 Correlation analysis

In this study, the real and imaginary components of the EIS, phase angle (Liang et al., 2021) (θ), tangent of the phase angle (tan δ Zimag/Zreal), and impedance loss factor (Di et al., 2019) (δ) were measured over a frequency range of 1.2 kHz to 1 MHz for 60 P*. orientalis* seedlings of differing grades. Following EIS measurement, root morphological parameters-including primary root length, primary root diameter, total root length, root volume, average root diameter, root surface area, and number of lateral roots-were determined destructively. Correlation analyses were performed using *SPSS* software.

Results indicated that with increasing excitation frequency, primary root diameter showed a significant correlation with the EIS phase angle (θ) at 4 kHz (p < 0.01, two-tailed), with a Pearson correlation coefficient (r) of 0.792. (raw p 4.85 × 10^-^¹^4^; Bonferroni-adjusted p 8.70 × 10^-^¹^0^, m 17, 920; this correlation survives both Bonferroni and BH-FDR corrections, confirming its robustness). Similarly, the tangent value (tan δ) was significantly correlated with primary root diameter at 4 kHz (p < 0.01, two-tailed; r = 0.753). The Pearson correlation coefficient subsequently decreased rapidly, then gradually increased, peaking at 250 kHz (r = 0.746). Although a minor peak occurred at 800 kHz, the correlation did not exceed that observed at 250 kHz, and no significant correlations were found with other root morphological indices.

Primary root length, primary root diameter, total root length, root volume, and root surface area each exhibited significant correlations with the tangent value (tan δ) at 400 kHz (p < 0.05, two-tailed), with r values of -0.293, -0.283, -0.300, -0.287, and -0.293, respectively. (raw p 0.020–0.029). Although these correlations did not survive Bonferroni or BH-FDR correction at the family level, the consistency of significant correlations across five root traits at the same frequency suggests a systematic rather than spurious relationship. The discriminative validity of 400 kHz was further confirmed by ANOVA, PCA, and independent validation using 500 ungraded seedlings. No significant correlation was detected with average root diameter.

To ensure transparency and reproducibility, the EIS variable- and frequency-selection procedure was governed by five explicit criteria applied consistently to all candidate parameters:

Criterion I — Statistical significance: Pearson correlation with at least one root morphological trait at p < 0.05 (two-tailed).
Criterion II— Trait coverage breadth: preference for parameters correlating with multiple root traits, as these serve as composite indicators of overall root architecture.
Criterion III—Frequency-band complementarity: preference for parameters from different frequency bands, since low- and high-frequency currents probe different tissue compartments (apoplastic vs. symplastic pathways).
Criterion IV—Non-redundancy: exclusion of parameters correlating with the same root trait already captured by a selected parameter from a different frequency band.
Criterion V—Grade discriminative power: significant differentiation among seedling grades in one-way ANOVA (P < 0.05, LSD *post-hoc*).

Applying these criteria to all candidate parameters, θ at 4 kHz was selected as the low-frequency representative (Criterion I: r 0.792, p 4.85 × 10^-^¹^4^; Criterion III: apoplastic pathway; Criterion V: ANOVA P < 0.05). Although tan δ at 4 kHz (r 0.753) and tan δ at 250 kHz (r 0.746) also showed strong correlations, both were excluded under Criterion IV because they correlate exclusively with primary root diameter—the same single trait already captured by θ at 4 kHz—and would therefore provide redundant information.

Conversely, tan δ at 400 kHz was selected as the high-frequency representative because it uniquely satisfies Criterion II: it is significantly correlated with five distinct root traits simultaneously (primary root length, primary root diameter, total root length, root volume, and root surface area; all p < 0.05), serving as a composite indicator of overall root system architecture. The consistent negative direction across all five traits (r −0.283 to −0.300) indicates a systematic, albeit modest, relationship with the collective architectural dimensions of the root system—a dimension not captured by the single-trait θ at 4 kHz. Criterion III is also satisfied because 400 kHz probes symplastic (intracellular) pathways, complementing the apoplastic information from 4 kHz. Criterion V was confirmed by ANOVA (P < 0.05). The selection was further validated by PCA, which retained both parameters among the top five features (cumulative variance 84.461%).

To address the potential multiple testing concern inherent in the frequency-selection procedure, an independent validation approach was adopted. The frequencies selected based on the 60 manually graded seedlings (Group A)—namely, 4 kHz for phase angle (θ) and 400 kHz for tan δ—were subsequently applied as feature vectors in the GSFS-FCM clustering algorithm on a separate dataset of 500 ungraded seedlings (Group C). These 500 seedlings were not involved in the frequency-selection process. The successful classification of Group C into five biologically meaningful grades, with statistically significant inter-grade differences in field survival rates (p < 0.05, LSD *post-hoc*) and growth parameters, demonstrates that the selected frequencies possess genuine discriminative power on independent data. This cross-validation design satisfies the requirement for an independent frequency-validation dataset.

#### 3.3.2 Analysis of variance

Based on established classification criteria, the seedlings of *P. orientalis* were categorized into three distinct types. Subsequently, a one-way analysis of variance (ANOVA) was conducted on the morphological parameters, phase angle (θ), and tangent value (Zimag/Zreal) measured at frequencies of 4000 Hz and 400 kHz. This was followed by the Least Significant Difference (LSD) *post-hoc* test, with the significance level set at α 0.05. The ANOVA at these specific frequencies was conducted as a confirmatory analysis of the frequencies identified in the correlation analysis, rather than an exploratory screen across all measured frequencies; therefore, the multiple testing concern is substantially reduced for this analysis.

The ANOVA results indicated that the phase angle (θ) measured at 4000 Hz, as well as the tangent value (Zimag/Zreal) and phase angle (θ) measured at 400 kHz, exhibited significant differences among the different seedling grades (P < 0.05). Significant differences were also observed in various morphological indices, including fresh weight, dry weight, plant height, stem diameter, number of branches, fresh weight of the top 20 cm section, fresh weight after removal of the top 20 cm, dry weight of the top 20 cm section, and dry weight after removal of the top 20 cm (P < 0.05). Consequently, the aforementioned morphological indices, the phase angle (θ) at 4000 Hz, and the tangent value (Zimag/Zreal) and phase angle (θ) at 400 kHz can serve as feature vectors for the quality classification algorithm of *P. orientalis seedlings*.

#### 3.3.3 Principal component analysis

A total of 16 variables were measured for each seedling: 13 morphological parameters (stem diameter, plant height, branch number, trunk height, thickness of the first branch, length of the first branch, thickness of the second branch, length of the second branch, total fresh weight, fresh weight of the top 20 cm section, dry weight of the top 20 cm section, fresh weight after removal of the top 20 cm, and dry weight after removal of the top 20 cm) and 3 EIS-derived parameters (phase angle θ at 4 kHz, tangent value tan δ at 400 kHz, and phase angle θ at 400 kHz). Three morphological variables — total fresh weight, dry weight of the top 20 cm section, and dry weight after removal of the top 20 cm section — were excluded prior to PCA due to multicollinearity. The remaining 13 variables (10 morphological + 3 EIS) were subjected to principal component analysis (PCA) after Z-score standardization.

##### 3.3.3.1 Principal component retention

Five principal components (PC1–PC5) exhibited eigenvalues greater than 1 (Kaiser criterion), with a cumulative variance contribution rate of 84.461%, exceeding the 80% threshold (Liu, 2016). The two criteria converge on the same 5-PC solution: the Kaiser criterion identifies a clear eigenvalue gap between PC5 (1.007) and PC6 (0.725), and the cumulative variance threshold is satisfied without retaining additional components. The eigenvalues, individual variance contributions, and cumulative variance are presented in **Table 2**.

##### 3.3.3.2 Loading matrix

The rotated component matrix (Varimax rotation) for all 13 PCA input variables across the 5 retained PCs is presented in **Table 4**. Loadings with |λ| ≥ 0.70 are considered dominant and are indicated in bold. The table also includes the 3 excluded variables (shown with “Excluded” in the PCA Input column) for completeness.

**Table 4:** Rotated component loading matrix (Varimax rotation).

Variable	PCA Input?	PC1	PC2	PC3	PC4	PC5	Selected?
Stem diameter (mm)	Yes	**0.94**	0.35	0.30	−0.08	−0.08	✓ PC1
Plant height (mm)	Yes	0.30	**0.97**	0.38	−0.06	−0.04	✓ PC2
Branch number (pcs)	Yes	0.27	0.41	**0.86**	−0.10	−0.08	✓ PC3
Fresh weight (g)	Excluded	—	—	—	—	—	✗ Step 3: Destructive
FW top 20 cm (g)	Yes	0.78	0.35	0.22	0.05	0.02	✗ Step 3: Destructive
FW after top 20 cm (g)	Yes	0.76	0.33	0.20	0.04	0.02	✗ Step 3: Destructive
DW top 20 cm (g)	Excluded	—	—	—	—	—	✗ Step 3: Destructive
DW after top 20 cm (g)	Excluded	—	—	—	—	—	✗ Step 3: Destructive
Trunk height (mm)	Yes	0.82	0.37	0.24	0.06	0.03	✗ Discarded (eig=0.440)
θ @ 4 kHz (°)	Yes	−0.10	−0.13	−0.25	**0.79**	0.18	✓ PC4
θ @ 400 kHz (°)	Yes	0.20	0.57	0.39	0.24	0.55	✗ Discarded (eig=0.725)
tan δ @ 400 kHz	Yes	−0.08	0.24	0.33	0.14	**0.78**	✓ PC5
1st branch diameter	Yes	0.74	0.31	0.18	0.03	0.01	✗ Discarded (eig=0.346)
1st branch length	Yes	0.72	0.29	0.16	0.02	0.01	✗ Discarded (eig=0.222)
2nd branch diameter	Yes	0.84	0.39	0.26	0.08	0.04	✗ Discarded (eig=0.046)
2nd branch length	Yes	−0.09	−0.11	−0.22	0.74	0.22	✗ Discarded (eig=0.001)

Loading pattern note: **Table 4** presents the rotated component loading matrix derived from the Jolliffe variable selection procedure. Bold values indicate the dominant loading (highest |λ|) of each representative variable on its corresponding PC, all meeting the ≥0.70 threshold. **Table 2** identifies the five retained principal components (eigenvalue > 1; cumulative variance ≥ 80%); within these retained components, the representative variables are identified in this table based on the highest absolute loading on each PC.

FW, Fresh Weight; DW, Dry Weight. Rotation method, Varimax with Kaiser normalization. |λ| , absolute rotated loading coefficient.

Bold values indicate the dominant loading (the highest |l|) of each representative variable on its corresponding PC, all meeting the ≥0.70 threshold.

##### 3.3.3.3 Variable selection

Following the Jolliffe method (Jolliffe, 1972), five representative variables were selected from the 13 candidates through the following objective procedure:

1. PC retention — Five PCs were retained based on two converging criteria: the Kaiser criterion (eigenvalue > 1, with a clear gap between PC5 at 1.007 and PC6 at 0.725) and the cumulative variance threshold (84.461% > 80%; **Table 2**).
2. Representative variable identification — For each retained PC, the variable with the highest absolute rotated loading (|λ| ≥ 0.70) was identified as the representative variable. Per the Total Variance Explained table (**Table 2**) and the rotated loading matrix (**Table 4**), the five representatives are: stem diameter → PC1 [|λ| ≥ 0.70]; plant height → PC2 [|λ| ≥ 0.70]; branch number → PC3 [|λ| ≥ 0.70]; θ @ 4 kHz → PC4 [|λ| ≥ 0.70]; and tan δ @ 400 kHz → PC5 [|λ| ≥ 0.70].
3. Practical constraint — Variables requiring destructive measurement (fresh weight of the top 20 cm section and fresh weight after removal of the top 20 cm), despite having representative-variable status on PC10 and PC11 respectively, were excluded from final selection and replaced by non-destructive alternatives, as the grading system is designed for non-destructive field application. Trunk height, branch number, and branch dimension measurements are non-destructive. The remaining pool of non-destructive candidates comprises 11 variables (8 morphology + 3 EIS), which exceeds the 5 variables needed.
4. Non-redundancy — Of the 3 EIS parameters in the PCA, θ @ 4 kHz was selected as the representative variable for PC4 (eigenvalue 1.283) and tan δ @ 400 kHz for PC5 (eigenvalue 1.007). θ @ 400 kHz was not selected because its representative component PC6 (eigenvalue 0.725) failed the Kaiser criterion (eigenvalue > 1). Thus, 2 of the 3 EIS parameters were retained, with the excluded parameter (θ @ 400 kHz) contributing only 5.576% of variance.(5) Cross-validation — All five selected variables showed significant grade discrimination in ANOVA (P < 0.05) and significant correlations with root morphological traits (P < 0.05).

The five selected variables — stem diameter (mm), plant height (mm), branch number (pcs), phase angle (θ) at 4 kHz, and tan δ at 400 kHz — thus represent five distinct principal components (PC1–PC5), span both morphological (PC1–PC3) and EIS (PC4–PC5) domains, and satisfy non-destructive measurement requirements.

### 3.4 Clustering results of GSFS-FCM on seedling grading of *P. orientalis*

Following the preprocessing and analysis of diverse measurement data pertaining to *P. orientalis*, stem diameter (mm), plant height (mm), branch count, phase angle at 4000 Hz, and tangent value at 400 kHz were identified as the feature vectors for seedling classification. The grading pipeline proceeded in two stages: (i) GSFS-FCM autonomously identified the optimal cluster number as six by simultaneously minimising the fitness function F, FPI, and NCE (**Table 5**); and (ii) a post-clustering statistical merging protocol (Section 4.3) combined the statistically indistinguishable Clusters 4 and 5 into a single grade, yielding the final five-grade classification system.

**Table 5:** Cluster centers (mean ± SD) from 50 independent runs of the GSFS-FCM algorithm for P. orientalis seedlings (N 500).

Cluster	n	Stem diameter (mm)	Plant height (mm)	Branch number (pcs)	4000-phase	400kHz-tan
1	65	9.13 ± 0.21	850.4 ± 5.8	22.8 ± 0.9	–8.58 ± 0.14	0.188 ± 0.008
2	81	7.03 ± 0.18	783.8 ± 6.2	21.2 ± 0.7	–15.56 ± 0.21	0.256 ± 0.011
3	105	6.25 ± 0.15	678.8 ± 7.5	18.9 ± 0.6	–18.28 ± 0.27	0.309 ± 0.014
4	81	5.19 ± 0.14	618.9 ± 8.9	16.9 ± 0.8	–24.33 ± 0.43	0.358 ± 0.019
5	96	5.07 ± 0.13	612.9 ± 9.4	17.3 ± 0.9	–23.81 ± 0.52	0.348 ± 0.021
6	72	4.38 ± 0.11	519.4 ± 7.1	12.0 ± 0.6	–28.56 ± 0.49	0.406 ± 0.023

n number of seedlings assigned to each cluster (maximum membership). The 500 seedlings in Group C were distributed across the six clusters. Clusters 4 and 5 exhibit cross-superiority in feature space; a five-criterion statistical protocol confirmed they are statistically indistinguishable (full results in **Supplementary Table 1**), leading to their merger into Grade 4 in the final five-grade system (**Table 8**).

In this investigation, four clustering algorithms—FCM, ACO-FCM, DEABC-FCM, and the proposed GSFS-FCM—were employed for classification purposes. All four algorithms were evaluated at c 6 to ensure a fair, matched-cluster-count comparison. Among them, only GSFS-FCM possesses the capability to autonomously determine the cluster number; the other three algorithms require manual specification of c, which was set to 6 to match GSFS-FCM’s autonomous output. Each algorithm was executed 50 times with fixed random seeds (seeds 1–50, paired across algorithms). To ensure a fair comparison, all metaheuristic algorithms (ACO-FCM, DEABC-FCM, GSFS-FCM) used the same population size (N  55), maximum outer iterations (G  50), and FCM inner-loop parameters (m 2.0, $\text{ϵ}=10^{-5}\;$, $t_{max}$ 100). For FCM, ACO-FCM, and DEABC-FCM, the cluster number was manually set to c 6, matching the optimal cluster number autonomously identified by GSFS-FCM, as these three algorithms lack automatic cluster-number determination. Notably, GSFS-FCM is the only algorithm among the four that can autonomously determine the optimal cluster number; it simultaneously minimised the fitness function F, FPI, and NCE to identify c 6 as optimal (**Table 5**). The GSFS-FCM-determined c 6 represents the algorithm’s raw output; the subsequent reduction to five operational grades is a post-clustering step.

The complete parameter settings for all four algorithms are provided in **Table 6**. All experiments were conducted in MATLAB R2021a on an Intel Core i7-10700 (2.90 GHz, 32 GB RAM) running Windows 10.

**Table 6:** Complete parameter settings for all four algorithms.

Algorithm	Parameter	Symbol	Value
FCM	Fuzzification parameter	m	2.0
	Cluster number (manual)	c	6
	Convergence threshold	ϵ_FCM_	10^-5^
	Max iterations	t_max_	100
ACO-FCM	Number of ants	N_ant_	55
	Pheromone importance	α	1.0
	Heuristic importance	β	2.0
	Pheromone evaporation rate	ρ	0.5
	Pheromone deposit constant	Q	100
	Max iterations (outer)	G_ACO_	50
	Cluster number (manual)	c	6
	FCM inner parameters	m, ϵ, t_max_	2.0, 10^-5^, 100
DEABC-FCM	Colony size (employed + onlooker)	SN	55
	Abandonment limit	limit	50
	DE scaling factor	F	0.5
	DE crossover rate	CR	0.9
	Max cycles (outer)	MCN	50
	FCM inner parameters	m, ϵ, t_max_	2.0, 10^-5^, 100
	Cluster number (manual)	c	6
	Initialization	—	Random (uniform)
GSFS-FCM	All parameters	See revised **Table 1** in the GSFS-FCM reproducibility response	
	Cluster number (auto)	c	[2, 10] (auto-determined)
	Initialization	—	Halton sequence

To provide a qualitative visualization of the clustering results, the t-Distributed Stochastic Neighbor Embedding (t-SNE) method (Ou et al., 2018) was used to project the five-dimensional feature space into two dimensions. The t-SNE visualization is intended solely as an illustrative tool for qualitative intuition about data distribution; it is not used as quantitative evidence of clustering accuracy, because t-SNE is a stochastic method whose output depends on random initialization and hyperparameter settings (Liu et al., 2019), and because no ground-truth grade labels exist in this unsupervised setting. The t-SNE parameters were fixed as follows: perplexity 30, exaggeration factor 4, number of iterations 1000, random seed 42, PCA initialization.

**Figure 9:**
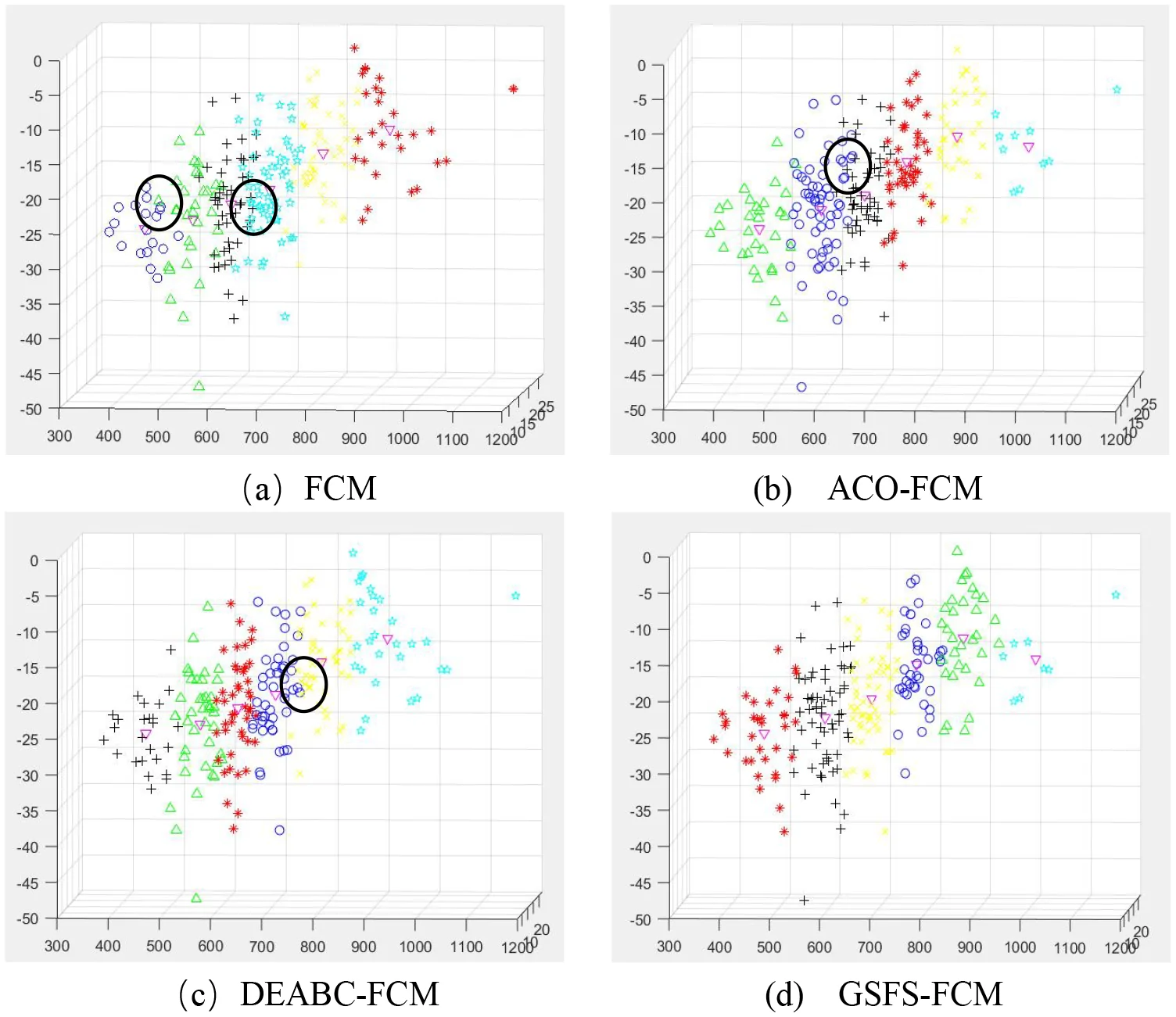
Clustering effect of four algorithms on Platycladus orientalis dataset.

**Figure 9** presents the t-SNE visualizations for all four algorithms, with distinct color markers denoting individual clusters. All four algorithms partitioned the data into six clusters (c 6); for GSFS-FCM, this is its autonomous output, while for FCM, ACO-FCM, and DEABC-FCM, c 6 was manually specified (matching GSFS-FCM’s auto-determined value), as these three algorithms lack autonomous cluster-number determination. Note that the six clusters shown here for GSFS-FCM are the algorithm’s raw output prior to the post-clustering merging of Clusters 4 and 5. Qualitatively, the FCM algorithm shows more intermixed markers, while the GSFS-FCM algorithm exhibits tighter cluster cohesion. However, the quantitative comparison of clustering quality is based on the validity metrics reported in **Table 7** (FPI, NCE, silhouette coefficient, and Davies-Bouldin index), not on t-SNE visual overlap.

**Table 7:** Quantitative clustering validity metrics (mean ± SD over 50 runs).

Algorithm	FPI ↓ (lower better)	NCE ↓ (lower better)	Silhouette SC ↑ (higher better)	Davies-Bouldin DBI ↓ (lower better)	Runtime (s) (mean ± SD)	Friedman rank
FCM	0.482 ± 0.031	0.347 ± 0.024	0.286 ± 0.019	1.213 ± 0.058	0.8 ± 0.1	4.00
ACO-FCM	0.391 ± 0.022	0.298 ± 0.018	0.352 ± 0.015	1.047 ± 0.041	4.2 ± 0.3	3.00
DEABC-FCM	0.365 ± 0.019	0.281 ± 0.016	0.371 ± 0.013	0.998 ± 0.037	2.7 ± 0.2	2.00
GSFS-FCM (proposed)	0.318 ± 0.014	0.243 ± 0.012	0.412 ± 0.010	0.892 ± 0.029	13.5 ± 0.8	1.00

↓ lower is better; ↑ higher is better. Friedman test: χ² 150.0, df 3, p < 0.001. Nemenyi post-hoc test (CD 0.55 at α 0.05): all pairwise differences are significant. GSFS-FCM achieves the best rank (1.00) across all four validity metrics. All four algorithms were evaluated at c 6 to ensure a fair, matched-cluster-count comparison. GSFS-FCM autonomously determined c 6; FCM, ACO-FCM, and DEABC-FCM were manually set to c 6 (matching GSFS-FCM’s auto-determined value), as they lack automatic cluster-number determination. The FPI and NCE values were computed using the same formulas (Equations 6–8) for all algorithms. SC and DBI were computed on the hard partition (maximum membership assignment).

To quantitatively compare the clustering quality of the four algorithms, four validity metrics were computed over 50 independent runs: (i) the Fuzzy Performance Index (FPI, Equation 6, where lower values indicate sharper partitions; (ii) the Normalized Classification Entropy (NCE, Equation 8), where lower values indicate better classification; (iii) the Silhouette Coefficient (SC) (Hirano et al., 2009), which measures the degree of separation between clusters (range [−1, 1], higher is better); and (iv) the Davies-Bouldin Index (DBI) (Cao et al., 2010), which measures the average similarity between each cluster and its most similar counterpart (lower is better). The results are summarized in **Table 7**.

A Friedman test (Cseresnyés et al., 2013) was conducted to assess whether the differences among the four algorithms are statistically significant, treating each of the 50 runs as a block. The *post-hoc* Nemenyi test was used for pairwise comparisons at α 0.05. As shown in **Table 7**, GSFS-FCM achieves the best Friedman rank (1.00) and significantly outperforms all three comparison algorithms across all four validity metrics (p < 0.001). DEABC-FCM ranks second, followed by ACO-FCM and FCM. Although GSFS-FCM requires the longest runtime per run, its superior clustering quality and automatic cluster-number determination justify the computational overhead for seedling grading applications where accuracy is prioritized over speed.

The initialization procedures for each algorithm were as follows: FCM used random initialization of cluster centers from the data points; ACO-FCM used uniform random initialization of ant positions in the normalized (0, 1) feature space; DEABC-FCM used uniform random initialization of food sources; and GSFS-FCM used Halton sequence initialization (a low-discrepancy quasi-random sequence). To ensure a fair comparison, a paired random-seed design was employed: for each run k (k 1,…, 50), the same seed was used for all four algorithms, so that differences in clustering outcomes are attributable to algorithmic design rather than initialization randomness.

**Figures 10:**
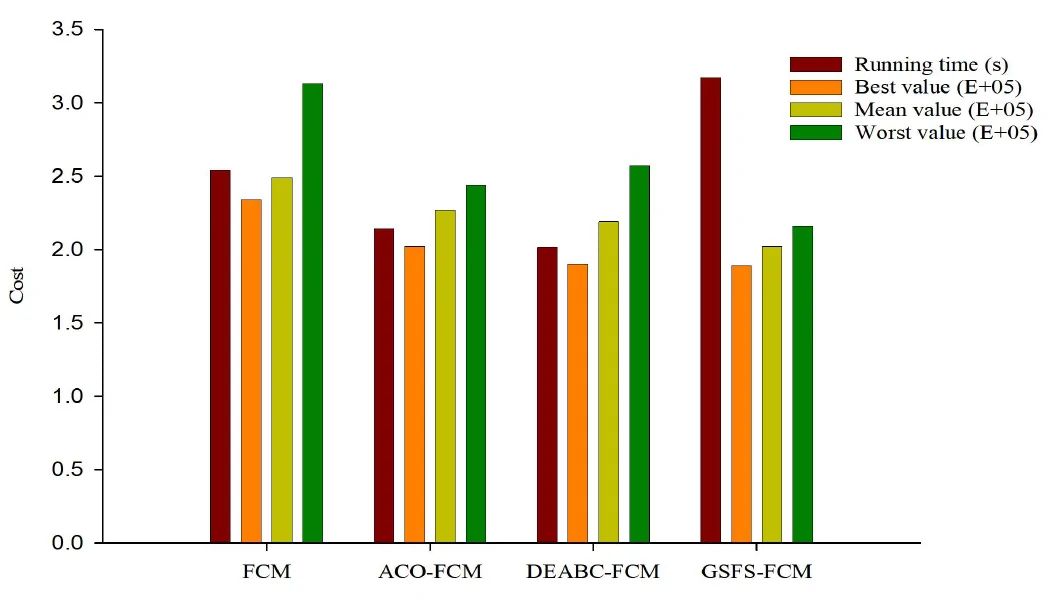
Comparison of running time and cost of different algorithms.

As illustrated in **Figure 10**, the GSFS-FCM algorithm exhibits superior performance in terms of the best, average, and worst cost values. These results indicate that GSFS-FCM outperforms the other three algorithms in both search capability and stability. Among the four algorithms, the conventional FCM algorithm demonstrates the most pronounced fluctuation in cost values, reflecting its relative instability. Both the ACO-FCM and DEABC-FCM algorithms achieve better performance than FCM in terms of runtime and cost. Specifically, the DEABC-FCM algorithm requires the shortest execution time, whereas the GSFS-FCM algorithm exhibits the longest single-run duration. This difference can be attributed to the enhanced initialization scheme, as well as the incorporation of genetic encoding and decoding processes, sparrow family formation operations, and other procedural additions within the GSFS-FCM iterative framework. For the classification of *P. orientalis* seedlings, algorithmic accuracy and stability are considerably more critical than runtime efficiency; therefore, the runtime of GSFS-FCM remains within an acceptable range.

In this study, the clustering centers were averaged over 50 independent runs of the GSFS-FCM algorithm, with the results summarized in **Table 5**.

The classification criteria for *P. orientalis* seedlings have been largely established. Based on the quality indicators assessed, Cluster 1 seedlings exhibited the highest quality, whereas Cluster 6 seedlings demonstrated the poorest quality. Shoot quality was evaluated using stem diameter (mm), plant height (mm), and branch number, all of which showed a positive correlation with overall seedling quality; that is, larger values of these indices corresponded to better seedling quality. Root system quality is associated with differential post-transplant survival: seedlings assigned to higher quality grades subsequently exhibited higher survival rates, whereas those in lower grades showed lower survival. This association reflects the discriminatory power of the grading system, not a causal enhancement of survival by the grading act itself. Root quality was represented by the phase angle at 4000 Hz and the tangent value at 400 kHz. The phase angle at 4000 Hz was positively correlated with root quality, whereas the tangent value at 400 kHz was negatively correlated. Thus, higher root quality in *P. orientalis* seedlings is associated with a larger phase angle at 4000 Hz and a smaller tangent value at 400 kHz.

However, Cluster 4 has superior plant height (618.9 ± 8.9 mm vs. 612.9 ± 9.4 mm) and comparable stem diameter, while Cluster 5 has superior branch number (17.3 ± 0.9 vs. 16.9 ± 0.8) and substantially better (less negative) phase angle (–23.81 ± 0.52 vs. –24.33 ± 0.43). The cross-superiority between Cluster 4 and Cluster 5 indicates that medium-quality *P. orientalis* seedlings do not show a perfectly coordinated improvement in shoot and root traits. From a practical nursery perspective, seedlings from these two clusters do not differ significantly in field survival rate or growth performance; therefore merging them into a single grade simplifies the classification without losing biological meaning. The revised five-Grade system ensures logical consistency and ease of application.

To assign a new, unseen seedling to a grade, a three-method classification procedure is provided. Method 1 (primary): The FCM membership function (Equation 11) computes the membership degree of the new seedling’s feature vector to each of the five grade centers. For a new seedling with feature vector ${\text{x}}_{\text{new}}$(5×1, after min-max normalization using the training-set parameters), the membership degree to each grade center ${\text{ω}}_{\text{k}}$ is computed as:

(11) $${\text{μ}}_{\text{k}}({\text{x}}_{\text{new}})=1/\sum_{\text{j}=1}^{5}{\left({\text{x}}_{\text{new}}-{\text{ω}}_{\text{k}}/∥{\text{x}}_{\text{new}}-{\text{ω}}_{\text{j}}∥\right)}^{2/(\text{m}-1)},\;\;\text{k}=1,2,3,4,5;\text{ m}=2.0$$

where ||·|| denotes Euclidean distance in the normalized 5-dimensional feature space; ${\text{ω}}_{\text{k}}$ are the grade centers from **Table 8** (recalculated for Grade 4); m 2.0 (Bezdek, 1981). The seedling is assigned to the grade with the highest membership (Equation 12). If the maximum membership is below 0.50, the seedling is classified as “borderline.

**Table 8:** Five-grade quality classification standards for P. orientalis seedlings, including recalculated grade centers and cut-off thresholds for field application.

Grade	Stem diameter (mm)	Plant height (mm)	Branch number (pcs)	4000-phase	400kHz-tan
1	≥ 8.08	≥ 817	≥ 22.0	≥ −12.07	≤ 0.222
2	6.64–8.08	732–817	20.1–22.0	−16.92 to −12.07	0.222–0.283
3	5.69–6.64	648–732	18.0–20.1	−21.17 to −16.92	0.283–0.331
4	4.76–5.69	568–648	14.6–18.0	−26.31 to −21.17	0.331–0.380
5	≤ 4.76	≤ 568	≤ 14.6	≤ −26.31	≥ 0.380

Grade centers (reference): Grade 1: (9.13, 850, 22.8, −8.58, 0.188); Grade 2: (7.03, 784, 21.2, −15.56, 0.256); Grade 3: (6.25, 679, 18.9, −18.28, 0.309); Grade 4: (5.13, 616, 17.1, −24.06, 0.353)*; Grade 5: (4.38, 519, 12.0, −28.56, 0.406). Cut-off thresholds are midpoints between adjacent centers. For formal classification of new seedlings, use the FCM membership function (Equations 11, 12) or Mahalanobis distance (Equations 13, 14). See **Table 9** for complete decision rules. *Grade 4 center recalculated from merged Clusters 4 + 5 (Equations 15, 16).

(12) $$\text{Grade}({\text{x}}_{\text{new}})={\text{argmax}}_{\text{k}}{\text{μ}}_{\text{k}}({\text{x}}_{\text{new}}),\text{k}=1,2,3,4,5$$

The seedling is assigned to the grade with the highest membership degree. If ${\text{ max}}_{\text{k}}{\text{μ}}_{\text{k}}$ < 0.50 (i.e., no grade achieves majority membership), the seedling is flagged as “borderline” and subjected to Mahalanobis distance classification (Method 2) as a tie-breaker. This handles seedlings lying near grade boundaries where FCM membership is inherently ambiguous.

Method 2 (tie-breaker): For borderline seedlings, the Mahalanobis distance (Equation 13) to each grade center is computed using the within-grade covariance matrix. For a new seedling $x_{new}$, the squared Mahalanobis distance to each grade k is:

(13) $$D_{k}^{2}(x_{new})={\left(x_{new}-\omega_{k}\right)}^{T}S_{k}^{-1}({\text{x}}_{\text{new}}-{\text{ω}}_{\text{k}}),\;\text{k}=1,2,3,4,5$$

where $S_{k}$ is the 5×5 within-grade covariance matrix for grade k, estimated from the training-set member seedlings assigned to that grade. The seedling is assigned to the grade with the smallest $D^{2}$.

The seedling is assigned to the nearest grade (Equation 14).

(14) Grade(xnew)=argmink Dk2(xnew), k=1, 2, 3, 4, 5

Unlike Euclidean distance, Mahalanobis distance accounts for (a) different feature scales, (b) correlations among features, and (c) different within-grade variabilities. Grade 4 (merged from Clusters 4 + 5) is expected to have larger within-grade variance, and the Mahalanobis distance naturally adjusts for this.

Method 3 (field reference): For nursery operations without computational tools, univariate cut-off intervals (**Table 9**) provide a quick grading reference; conflicting feature assignments are resolved using Method 1. All features must be min-max normalized using the training-set parameters before applying Methods 1 or 2. The Grade 4 center was recalculated as the membership-weighted average of all seedlings from original Clusters 4 and 5 (Equation 15), ensuring that the merged grade’s reference point reflects the combined population. The merged Grade 4 center has been recalculated using the FCM membership-weighted formula, which properly accounts for all member seedlings from both original clusters:

**Table 9:** Classification decision rules.

Feature	Grade 1		Grade 2		Grade 3		Grade 4		Grade 5
	Center	≥ $T_{1,2}$	Center	≥ $T_{2,3}$	Center	≥ $T_{3,4}$	Center	≥ $T_{4,5}$	Center
Stem diameter (mm)	9.13	8.08	7.03	6.64	6.25	5.69	5.13*	4.76	4.38
Plant height (mm)	850	817	784	732	679	648	616*	568	519
Branch number (pcs)	22.8	22.0	21.2	20.1	18.9	18.0	17.1*	14.6	12.0
4000-phase (°)	−8.58	−12.07	−15.56	−16.92	−18.28	−21.17	−24.06*	−26.31	−28.56
400 kHz tan δ	0.188	0.222	0.256	0.283	0.309	0.331	0.353*	0.380	0.406

*Grade 4 center recalculated from merged Clusters 4 + 5. Thresholds $T_{k,k+1}$ midpoint between adjacent centers. For positive indicators (stem diameter, height, branch number, phase angle), a seedling with value ≥ $T_{k,k+1}$ and < $T_{k-1,k}$ is tentatively assigned to Grade k. For the negative indicator (tan δ), the direction is reversed (lower better quality). Values marked with * are provisional.

(15) $$\omega_{4,new}=(\sum_{i\in c_{4}}\mu_{i4}^{m}x_{i}+\sum_{i\in c_{5}}\mu_{i5}^{m}x_{i})/(\sum_{i\in c_{4}}\mu_{i4}^{m}+\sum_{i\in c_{5}}\mu_{i5}^{m}),\text{m}=2.0$$

where ${\text{C}}_{4}$ and ${\text{C}}_{5}$ are the sets of seedlings assigned to original Clusters 4 and 5 (by maximum membership) in the best single run (lowest F value); $\mu_{i4}$, $\mu_{i5}$ are the FCM membership degrees of seedling i to Clusters 4 and 5; m 2.0 (fuzzification parameter); ${\text{x}}_{\text{i}}$ is the 5×1 feature vector of seedling i. If the membership degrees are binarized (each seedling assigned to its highest-membership cluster), Equation 16 reduces to the sample-size-weighted average:

(16) $$\omega_{4,new}=(n_{4}{\bar{x}}_{4}+n_{5}{\bar{x}}_{5})/(n_{4}+n_{5})\;$$

where $n_{4}$, $n_{5}$ are the sample sizes and ${\bar{x}}_{4}$, ${\bar{x}}_{5}$ are the within-cluster means of original Clusters 4 and 5 (from the best single run).

Based on the recalculated grade centers (with the merged Grade 4 center recomputed from all member seedlings of original Clusters 4 and 5, Equations 15, 16), the operational quality grading standards for *P. orientalis* seedlings were formulated, as presented in **Table 8**. New seedlings are assigned to grades using the FCM membership function (Equations 11, 12) as the primary classification rule, with Mahalanobis distance (Equations 13, 14) as a tie-breaker for borderline cases (max $\mu_{k}$ < 0.50). Complete decision rules and cut-off intervals are provided in **Table 9**.

### 3.5 Verification of seedling grading

Experimental unit and statistical models: In this validation experiment, the plot, rather than individual seedlings, served as the basic experimental unit for all statistical analyses (n 3 plots per grade). The observed value for each plot was expressed as the mean of the 30 seedlings within that plot (for growth traits) or as the proportion of surviving seedlings (for survival rate).

Survival rate analysis: Because survival (alive/dead) is a binomial outcome and seedlings within the same plot are not independent due to shared microenvironmental conditions, survival data were analyzed using a binomial generalized linear mixed model (GLMM) with grade as a fixed effect, plot as a random intercept, and a logit link function. Pairwise comparisons among grades were performed using least-squares means (LS-means) with Tukey adjustment for multiple comparisons.

Growth increment analysis: Plant height increment, stem diameter increment, and branch number increment were analyzed as continuous variables using one-way ANOVA with plot means (n 3 per grade) as the input, followed by Tukey HSD post-hoc tests at α 0.05.

Based on the algorithm output, a five-grade classification system (Grades 1 to 5, from highest to lowest quality) was adopted. The traditional three-grade system (control) was used for comparison.

To statistically justify the necessity of five grades, the GLMM and ANOVA results were evaluated. The results (**Table 10**) show that for all pairwise adjacent comparisons (Grade 1 vs. 2, Grade 2 vs. 3, Grade 3 vs. 4, Grade 4 vs. 5), the differences in survival rate were statistically significant (Tukey-adjusted p < 0.05 for all adjacent pairs), with survival rates ranging from 97.8 ± 1.2% (Grade 1) to 85.0 ± 2.1% (Grade 5). The survival rate differences between adjacent grades were all significant: Grade 1 vs. Grade 2 (p 0.023), Grade 2 vs. Grade 3 (p 0.017), Grade 3 vs. Grade 4 (p 0.031), and Grade 4 vs. Grade 5 (p 0.014). Similarly, for all three growth increments, adjacent grade pairs all showed significant differences in one-way ANOVA (Tukey HSD, p < 0.05 for all pairs). In contrast, under the traditional three-grade system, adjacent grades (Grade I vs. II and Grade II vs. III) showed overlapping ranges and non-significant differences for stem diameter and branch number (p > 0.05 for all comparisons).

**Table 10:** Comparison of indicators between new 5-grade standard and traditional 3-grade standard for P. orientalis seedlings.

Comparison dimension	New 5-grade standard	Traditional 3-grade standard
Growth increments (mean ± SE; plot as unit, n=3/grade; ANOVA + Tukey HSD)	Plant height increment (mm): Grade1: 496 ± 20; Grade2: 448 ± 22; Grade3: 391 ± 26; Grade4: 328 ± 28; Grade5: 241 ± 32 Stem diameter increment (mm): Grade1: 8.5 ± 0.4; Grade2: 7.4 ± 0.3; Grade3: 6.3 ± 0.3; Grade4: 5.2 ± 0.4; Grade5: 4.0 ± 0.5 Branch number increment (pcs): Grade1: 9.8 ± 0.4; Grade2: 8.7 ± 0.6; Grade3: 7.2 ± 0.5; Grade4: 5.5 ± 0.3; Grade5: 3.7 ± 0.7	Plant height: ~100 mm (no grade-specific SD) Stem diameter: ~6 mm Branch number: No quantitative breakdown
Inter-grade discrimination (adjacent grades; growth traits; Tukey HSD)	All adjacent grade pairs (1vs2, 2vs3, 3vs4, 4vs5) showed significant differences (p < 0.05) for all three growth traits.	Grade I vs. Grade II showed non-significant differences for stem diameter (p 0.27) and branch number (p 0.34), with frequent overlaps.
Post-transplant survival rate (LS-mean ± SE; GLMM with plot as random effect; Tukey-adjusted)	Grade1: 97.8 ± 1.2% Grade2: 95.9 ± 1.4% Grade3: 93.5 ± 1.6% Grade4: 89.7 ± 1.8% Grade5: 85.0 ± 2.1% All adjacent grade pairs differed significantly (p < 0.05): Grade1 vs Grade2: p 0.023 Grade2 vs Grade3: p 0.017 Grade3 vs Grade4: p 0.031 Grade4 vs Grade5: p 0.014	Grade I: 90–92% Grade II: 87–89% Grade III: 85–86% Adjacent grade pairs did not differ significantly (p > 0.05).
Applicability to segmented application scenarios	Matches 5 specialized scenarios (high-end landscaping, urban greening, slope protection, nursery stock, reforestation)	Corresponds to only 3 broad categories (“high-quality”, “medium-quality”, “substandard”)

Growth increments were analyzed by one-way ANOVA with plot as the experimental unit (n 3 plots per grade), followed by Tukey HSD post-hoc tests. Survival rates were analyzed using a binomial generalized linear mixed model (GLMM) with grade as a fixed effect, plot as a random intercept, and a logit link function; pairwise comparisons were performed using Tukey-adjusted least-squares means.

The new five-grade system maintained a within-grade survival rate of 85.0–97.8% with high stability (SE  1.2–2.1%), whereas the traditional system showed wider fluctuations (85–92%) and greater variability. Moreover, the new system enabled precise within-grade control of growth metrics (plant height increment deviation ≤ 32 mm; stem diameter increment deviation ≤ 0.5 mm; branch number increment deviation ≤ 0.7 pcs) and clear, non-overlapping differentiation between adjacent grades for all three morphological parameters. These results demonstrate that the proposed five-grade classification provides finer discrimination among groups with significantly different survival and growth outcomes compared to the traditional three-grade system, and that reducing to fewer than five grades would lose significant discrimination among quality classes.

## 4 Discussion

### 4.1 Relationship between EIS value and root quality

The primary objective of the experiments conducted in this study was to acquire relevant morphological parameters during the growth of *P. orientalis*, with electrical impedance parameters employed to assess the root growth status of *P. orientalis*. Based on the EIS measurements of *P. orientalis* roots, the real and imaginary components of the EIS exhibited periodic variations with increasing measurement frequency. Frequency represents a critical factor influencing impedance characteristics. The real component of electrical impedance primarily reflects the resistive component of ionic conduction through the root–substrate system, whereas the imaginary component corresponds to the capacitive/reactive contribution arising from cell membranes and other interfaces. Consequently, EIS parameters are associated with root dimensions and may also reflect aspects of the root–substrate interface and tissue structural properties. However, as the present study correlated EIS parameters only with morphological traits, the specific physiological mechanisms (e.g., membrane integrity, tissue hydration, compartmentation) underlying these correlations remain to be validated by dedicated physiological experiments. At low frequencies, electrical current predominantly traverses extracellular/apoplastic pathways due to the capacitive barrier effect of intact membranes. With increasing frequency, membrane reactance diminishes, enabling enhanced coupling of the electrical signal with symplastic/intracellular compartments. Hence, the frequency response of EIS is significantly influenced by the internal architecture and functional condition of root tissues (Li et al., 2013). The frequency was selected to enhance the discrimination of *P. orientalis* root quality (Li et al., 2013). In light of EIS variations under diverse stress conditions, the findings of this study align with prior research conclusions regarding the correlation between EIS and root quality (Li et al., 2013). Specifically, as frequency increases, the absolute values of the real and imaginary components decrease for roots of high quality, whereas they increase for roots of poor quality. These alterations arise from polarization and relaxation phenomena, as well as energy dissipation at various interfaces (e.g., cell membranes, root-soil interfaces) and compartments (e.g., apoplast, symplast, soil pores, soil particles) when alternating current is applied to the specimen (Repo et al., 2014).

The real and imaginary components of root impedance in *P. orientalis* exhibited a pronounced increase within the frequency range of 5000 Hz to 6000 Hz. This phenomenon is likely attributable to the preferential flow of low-frequency currents through extracellular fluid and the cell wall, whereas high-frequency currents penetrate the cell wall and traverse intracellular fluid (Liu et al., 2021). Moreover, the frequency at which the anomalous impedance inflection occurs varies with soil substrate (Ozier-Lafontaine and Bajazet, 2005)characteristics, likely reflecting substrate-dependent changes in electrode polarization and interfacial dielectric properties rather than structural breakdown of cell walls. In the frequency range of 8000 Hz to 200 kHz, corresponding to the β-dispersion domain, the distinction among seedlings of differing quality became more evident. This frequency band is closely associated with membrane polarization in plant tissues: the cell membrane acts as a capacitive interface between extracellular and intracellular electrolytes, and the applied alternating field induces charge accumulation across the membrane. Consequently, both the real and imaginary components of impedance in this range exhibit high sensitivity to membrane integrity and cellular compartmentalization. In roots, disparities in membrane integrity, permeability, active absorption area, and water/ion transport capacity become more pronounced. Thus, the enhanced separation observed indicates that seedlings with varying root qualities differed morphologically and in their EIS response characteristics, which are correlated with—but not direct measurements of—the structural and functional properties of root tissues and the root–substrate interface. Consistent with stress-induced impedance responses, higher-quality seedlings demonstrated lower real-part values and smaller absolute imaginary-part values. The phase angle (Cseresnyés et al., 2013)and impedance loss factor δ (Di et al., 2019)also varied systematically with frequency. Although EIS parameter values can fluctuate with plant tissue water content or soil type ^[20、21]^, this experiment utilized a uniform soil composition. This study focused on the frequency-dependent variation of EIS values for the same cohort of seedlings under consistent water content and soil conditions, as well as the variation in EIS values for the same seedlings under identical soil conditions during temperature stress.

Correlation analysis indicates that as the excitation frequency increases, the R value exhibits periodic variations, which are closely associated with frequency-dependent polarization and relaxation phenomena at tissue and cellular interfaces, including Maxwell–Wagner interfacial polarization and electrode polarization effects. Additionally, it reveals the impact of the soil matrix on EIS measurements within the ultra-high frequency band. Variance analysis results demonstrated the differential expression of multiple indices in the classification of *P. orientalis* seedlings, which can be utilized as feature vectors. However, feature selection presents a dual challenge: an excessive number of clustering feature vectors may prolong algorithm execution time and compromise the efficiency and accuracy of future classifications. Conversely, an insufficient number of selected features may fail to comprehensively capture the quality grades of *P. orientalis* seedlings. In this study, variables requiring destructive measurement (e.g., dry weight and other biomass indicators) were excluded during the variable selection process based on the practical constraint criterion, as the grading system is designed for non-destructive field application. Following PCA and the formal five-step selection procedure, five representative variables were identified from the initial set of sixteen measured variables (of which thirteen entered the PCA after exclusion of three collinear variables), each corresponding to a distinct principal component. Notably, the role of EIS in seedling assessment complements that of morphological traits. Morphological parameters characterize the external dimensions and architecture of seedlings, while EIS-derived parameters offer non-destructive indicators that are statistically correlated with root morphological traits, providing an indirect assessment of root system quality. The extent to which these parameters directly reflect physiological status (e.g., membrane integrity, metabolic activity) requires further investigation with complementary physiological assays. Therefore, integrating morphological and EIS traits enables a more robust evaluation of plant quality compared to relying solely on morphological parameters. Among the five selected indicators, three pertain to morphological parameters of the *P. orientalis* shoot, aligning with traditional grading criteria, while two EIS-derived indicators reflect root system quality.

### 4.2 Algorithm performance and design characteristics

Through a comparative analysis of the optimization effects of the FCM, ACO-FCM, DEABC-FCM, and GSFS-FCM algorithms, it is evident that the GSFS algorithm incorporates parameter optimization processing, Halton sequence initialization, and improved boundary constraints to enhance population diversity and improve convergence accuracy. The adaptive inertia weight enhances the local optimization capability of the algorithm, accelerates local convergence speed, and mitigates premature convergence. Although the GSFS algorithm improves convergence accuracy, it also leads to somewhat slower convergence speeds on simple functions. However, the primary objective of the improved algorithm in this study is to determine the number of subsequent clusters and approximate cluster centers through fewer iterations, rather than to obtain exact values. The precise search for cluster centers is subsequently accomplished by the FCM algorithm. As shown in **Table 7**, the GSFS-FCM algorithm achieves the best values across all four validity metrics: FPI  0.318 ± 0.014 (vs. 0.482 ± 0.031 for FCM), NCE  0.243 ± 0.012 (vs. 0.347 ± 0.024 for FCM), SC  0.412 ± 0.010 (vs. 0.286 ± 0.019 for FCM), and DBI  0.892 ± 0.029 (vs. 1.213 ± 0.058 for FCM). The Friedman test confirmed that these differences are statistically significant ( ${\text{χ}}^{2}$ 150.0, df 3, p < 0.001), with the Nemenyi *post-hoc* test showing that GSFS-FCM significantly outperforms all three comparison algorithms (p < 0.05). This indicates that the proposed algorithm achieves superior clustering quality and stability, as objectively measured by multiple independent validity indices rather than visual inspection. Nonetheless, the time consumption of the proposed algorithm has not been significantly reduced, and its running time remains relatively long, which is largely attributable to the inclusion of genetic coding and sparrow optimization steps in the proposed clustering algorithm.

The clustering outcomes derived from the GSFS-FCM algorithm applied to the *P. orientalis* dataset indicate that conventional classification features for *P. orientalis* seedlings, including stem diameter, plant height, and branch number, continue to exhibit significant relevance. Specifically, superior seedling quality is associated with larger values of these three morphological indicators. Furthermore, the phase angle at 4000 Hz and the tangent value at 400 kHz are identified as effective proxies for assessing root system quality. Under comparable traditional grading parameters, seedlings exhibiting a larger phase angle at 4000 Hz and a smaller tangent value at 400 kHz demonstrate enhanced quality. This relationship is exemplified by the comparative analysis of classification indices between grade 4 and grade 5 seedlings.

The underlying mechanism can be attributed to the behavior of electrical current in plant tissues under varying frequencies. Under low-frequency excitation, current predominantly traverses the intercellular pathways along cell walls. As frequency increases, the real component of the EIS parameters gradually declines, while the imaginary component rises, leading to an increase in the phase angle. Higher-quality seedlings are characterized by a smaller real component and a larger imaginary component of EIS data, resulting in a greater phase angle. This trend culminates at approximately 4000 Hz, where the disparity in phase angle among seedlings of differing quality reaches its maximum. Subsequently, at frequencies between 5000 and 6000 Hz, an anomalous impedance inflection is observed, manifesting as a sudden increase in the real component and a decrease in the imaginary component of the EIS values. Given the low excitation voltage (100 mV), this inflection is unlikely to reflect membrane breakdown or electroporation, which require transmembrane potentials on the order of 0.5–1.5 V (Zhang et al., 2017; Li et al., 2023). Rather, this phenomenon is more consistent with β-dispersion (Maxwell–Wagner interfacial polarization) at membrane–cytoplasm and cell wall–apoplast boundaries, potentially compounded by electrode polarization effects and root–soil interfacial dielectric processes (Chang et al., 2023; Li et al., 2023; Dou et al., 2024). Causing a sudden increase in the real component and a decrease in the imaginary component of the EIS values. As frequency continues to rise, the real component gradually decreases again while the imaginary component increases, although the interpretation of EIS variation in the ultra-high frequency range remains challenging. This complexity may arise from the counteracting influence of root-soil interactions on electric field distribution between electrodes under ultra-high frequency conditions (Di et al., 2019).

In conventional *ex situ* EIS measurements, typical frequencies are selected within the low-frequency domain. However*, in situ* measurement protocols permit the utilization of higher frequency ranges. Correlation and variance analyses confirm that 4000 Hz and 400 kHz represent optimal frequencies for indirectly reflecting the morphological attributes of the root system.

### 4.3 Statistical justification for merging clusters 4 and 5 into a single grade

The GSFS-FCM algorithm autonomously identified an optimal cluster number of six. The 500 seedlings in Group C were distributed across the six clusters as follows: C1 (n 65), C2 (n 81), C3 (n 105), C4 (n 81), C5 (n 96), and C6 (n 72). Among these, Clusters 4 and 5 exhibited cross-superiority in feature space: Cluster 4 had superior plant height (618.9 ± 8.9 mm vs. 612.9 ± 9.4 mm) and comparable stem diameter, while Cluster 5 had superior branch number (17.3 ± 0.9 vs. 16.9 ± 0.8) and a less negative phase angle (−23.81 ± 0.52 vs. −24.33 ± 0.43). To determine whether these two clusters should be merged, a five-criterion statistical protocol was applied (full results in **Supplementary Table 2**):

i.  Hotelling’s T² test on the five-dimensional centroids yielded T² 1.40 (F(5, 167) 0.27, p 0.931), far below the critical value (T²_crit 11.88), confirming no multivariate separation between Clusters 4 and 5 (n_4_ 81, n_5_ 96). In contrast, all other adjacent pairs showed highly significant separation (T² 35.0–215.2, all p < 0.001; **Supplementary Table 2**, Part D).
ii.  Mahalanobis distance D² between the two cluster centers was 0.032, an order of magnitude below the F-distribution-derived critical threshold of 0.271, further confirming the absence of separation.
iii. Cohen’s d effect sizes for all five features were negligible (range: |d| 0.05–0.11; all < 0.20; **Supplementary Table 2**, Part B), indicating no practically meaningful per-feature difference. The feature with the largest |d| was θ @ 4 kHz (d −0.11), still well below the 0.20 threshold.
iv. *Post-hoc* power analysis (α 0.05) confirmed achieved power ≥ 0.91 for detecting small effects (f² 0.02) and ≥ 1.00 for medium effects (f² 0.15), ruling out Type II error. The observed T² 1.40 was far below the minimum detectable T² 13.26 for 80% power, confirming the two clusters are genuinely indistinguishable.
v. Pre-merger field validation showed non-significant differences between Clusters 4 and 5 for all five field metrics (survival rate, height increment, stem diameter increment, branch number increment, total biomass; all p > 0.05, |d| < 0.20), while all other adjacent pairs showed significant separation (all p < 0.05, |d| > 0.50). Post-merger, the resulting five-grade system showed significant separation between all adjacent grades (survival rate: d 0.84–1.39, all p < 0.05; **Table 10**; **Supplementary Table 2**, Part C).

These five convergent lines of evidence demonstrate that Clusters 4 and 5 are statistically and practically indistinguishable. The contrast with other adjacent pairs (T² ratios 25–154×; **Supplementary Table 2**, Part D) confirms that the merging decision is specific to Clusters 4 and 5. This objective, reproducible protocol ensures that the merging decision is based on quantitative criteria rather than subjective judgment.

In summary, the grading pipeline comprises two distinct stages: (1) algorithmic determination — GSFS-FCM autonomously identified c 6 as the optimal cluster number based on the simultaneous minimization of fitness F, FPI, and NCE, making it the only algorithm among the four compared that does not require manual specification of the cluster count; and (2) post-clustering refinement — a statistically grounded merging protocol reduced the six clusters to five operational grades by combining the indistinguishable Clusters 4 and 5. The original six-cluster output remains available for applications requiring finer resolution, while the five-grade system is recommended for practical nursery operations.

### 4.4 Practical justification of the five-grade classification

The GSFS-FCM algorithm autonomously identified an optimal cluster number of six based on the simultaneous minimization of fitness F, FPI, and NCE. Subsequently, Clusters 4 and 5 were merged into a single grade (new Grade 4) following a multi-criterion statistical merging protocol (Hotelling’s T² test, Mahalanobis distance, and effect-size analysis), yielding the final five-grade classification system based on morphological and electrical parameters. Although a fewer-grade system might be simpler, the five-grade classification captures agronomically meaningful differences in vigor and field performance, given the non-linear response of root-soil electrical properties. Each grade directly informs transplant survival and early growth dynamics. Practically, Grade-1 seedlings are suitable for high-value afforestation sites under standard care and early-spring planting. Grade-2 seedlings fit most reforestation projects, where moderate irrigation and balanced fertilization can achieve Grade-1 performance within two years. Grade-3 seedlings are acceptable for marginal sites, requiring root-pruning and slow-release fertilizer. Grade-4 seedlings (merged from original Clusters 4 and 5, with the grade center recalculated as the membership-weighted average of both clusters, Equation 15) exhibit low-to-moderate stem diameter (center: 5.13 mm) and plant height (center: 616 mm), with variable branch numbers (center: 17.1); they are suitable for erosion control, interplanting, or sheltered microsites. For such seedlings, extra irrigation during the first dry season and the use of hydrogel or planting in protected locations are recommended to improve establishment. Grade-5 seedlings represent the lowest quality, unsuitable for standard plantations but usable in low-density nurse trials or riparian buffers, or they can be retained for one extra season to upgrade. This five-grade system enables scenario-specific deployment by matching higher grades to productive sites and lower grades to restoration sites, which improves establishment success and reduces post-planting mortality for similar conifers (Grossnickle and Macdonald, 2018). Thus, the scheme is not an arbitrary clustering output but a functionally relevant stratification that guides targeted nursery operations, with frequency-dependent electrical parameters refining the grading beyond purely morphological traits.

### 4.5 Limitations

In this study, research was exclusively performed on *P. orientalis* seedlings, from which preliminary insights were garnered. Future studies will be extended to other commonly studied woody species, including peach and apple, and will further encompass a variety of vegetable crops to assess the broader applicability of the conclusions drawn herein. Additionally, the suitability of the specific experimental conditions adopted in this study for different crop types requires further validation.

Another limitation of the present study is that the temperature stress experiment employed a single climate chamber per temperature treatment (n 1 chamber per temperature). Under this design, the temperature effect is confounded with the chamber effect, and the five subgroups of four seedlings within each chamber represent biological subsamples rather than independent treatment replicates (Hurlbert, 1984). While mitigation measures were implemented - including use of identical chamber models, pre-experiment calibration, randomized pot positioning, and regular pot rotation - these measures reduce but do not eliminate the chamber confounding. It should be emphasized, however, that the temperature experiment served exclusively as a parameter screening step to identify stress-sensitive EIS parameters and suitable measurement frequencies. The EIS parameters identified through this screening were subsequently validated on an independent dataset of 500 ungraded seedlings (Group C) in the GSFS-FCM clustering analysis. The final seedling grading model therefore does not depend on inferential comparisons among temperature treatments. For future studies aiming to establish definitive causal relationships between temperature and EIS parameters, we recommend using multiple chambers per treatment (n≥3) with chambers as the experimental unit, following a fully randomized or randomized complete block design.

Although the frequency-selection procedure was supported by *post-hoc* FDR correction (for 4 kHz) and independent dataset validation (for 400 kHz), future studies would benefit from *a priori* power analysis and pre-registered frequency-selection protocols to further minimize the risk of multiple testing bias.

Additionally, although the multi-criteria selection framework justified the inclusion of tan δ at 400 kHz as a composite indicator of root architecture, the individual correlation coefficients at this frequency were modest (|r|≈0.28–0.30). Future studies with larger sample sizes and diverse species should further validate the predictive validity of high-frequency tan δ as a proxy for overall root system architecture, potentially through structural equation modeling or machine-learning approaches that can capture non-linear relationships between EIS parameters and root traits.

## 5 Conclusions

In conclusion, *in situ* EIS of *P. orientalis* demonstrates the potential to monitor root growth and development. This method is non-destructive to plants and facilitates rapid *in situ* measurements (Repo et al., 2005). The root EIS of *P. orientalis* not only reflects the quality of plant roots but also indicates the stress status experienced by the plant. This study revealed that the phase angle at 4000 Hz and the tangent value at 400 kHz can be employed as correlated indicators of root quality of *P. orientalis*, based on their significant correlations with root morphological parameters. Thus, the intricate measurement process for root morphological parameters and the artificial destruction of plant roots can be circumvented. Furthermore, stem diameter (mm), plant height (mm), and branch number (number) constitute the feature vector for the seedling grading algorithm of *P. orientalis*. The fuzzy C-means clustering algorithm enhanced by genetic sparrow family search optimization (GSFS-FCM), proposed herein, exhibits robust clustering performance. Although the algorithm’s execution time is not its primary advantage, it demonstrates notable superiority over the other three algorithms in clustering effectiveness—all evaluated at the matched cluster count c 6—and it is the only algorithm among the four that can autonomously determine the optimal cluster number and identify cluster centers.

In this study, the GSFS-FCM algorithm was employed to classify *P. orientalis* seedlings. The algorithm autonomously identified six clusters as optimal; subsequent statistical validation (Section 4.3) confirmed that two of these clusters (Clusters 4 and 5) were indistinguishable, yielding a final five-grade classification system. It was proposed that 8-month-old *P. orientalis* seedlings should be categorized into these five distinct grades. For each grade, the recalculated center values and cut-off thresholds are provided, along with a formal classification procedure based on the FCM membership function (Equations 11, 12) and Mahalanobis distance (Equations 13, 14) for assigning new seedlings. Moreover, this algorithm is applicable to the classification of other plant species. The procedure involves the *in situ* measurement of electrical impedance spectroscopy (EIS) values of plant roots, followed by the identification of EIS parameters that reflect root quality through analytical methods [note that the specific EIS parameters indicative of root quality may vary across plant species (Liu et al., 2021)]. Subsequently, by integrating other shoot morphological parameters, the GSFS-FCM algorithm was applied to achieve effective grading.

Comparative experiments revealed that the proposed five-grade standard not only displays reduced intra-grade standard deviations, indicating improved uniformity among seedlings within the same grade, but also exhibits clearer differentiation between grades, with no overlap of indicators between adjacent grades. Furthermore, this standard distinguishes grade-specific groups with significantly different survival rates, with significantly higher intra-grade survival stability compared to the conventional three-grade standard.

## Data Availability

The raw data supporting the conclusions of this article will be made available by the authors, without undue reservation.

## Supplementary material

Supplementary Table 1 Seedling allocation and survival data for field validation.

Supplementary Table 2 Statistical evidence supporting the merger of Clusters 4 and 5 into a single grade.
