# Supplementary material for: Research on seedling grading of Platycladus orientalis based on fuzzy C-means clustering algorithm improved by genetic sparrow family search optimization
Source: Front Plant Sci. 2026 Sep 3;17:1929618. doi: 10.3389/fpls.2026.1929618 (PMC13582205; doi:10.3389/fpls.2026.1929618)
Supplement: Supplementary file 1 [file Table1.docx]

| **Table S1: Seedling allocation and survival data for field validation** | | | | | | |
| --- | --- | --- | --- | --- | --- | --- |
| System | Grade | Plot | Block | Seedlings planted (per plot) | Surviving seedlings (180 d) | Survival rate (%) |
| New 5-grade | 1 | 1 | I | 30 | 29 | 96.7 |
| New 5-grade | 1 | 2 | II | 30 | 30 | 100 |
| New 5-grade | 1 | 3 | III | 30 | 29 | 96.7 |
| New 5-grade | 2 | 1 | I | 30 | 28 | 93.3 |
| New 5-grade | 2 | 2 | II | 30 | 29 | 96.7 |
| New 5-grade | 2 | 3 | III | 30 | 29 | 96.7 |
| New 5-grade | 3 | 1 | I | 30 | 28 | 93.3 |
| New 5-grade | 3 | 2 | II | 30 | 27 | 90 |
| New 5-grade | 3 | 3 | III | 30 | 29 | 96.7 |
| New 5-grade | 4 | 1 | I | 30 | 27 | 90 |
| New 5-grade | 4 | 2 | II | 30 | 28 | 93.3 |
| New 5-grade | 4 | 3 | III | 30 | 26 | 86.7 |
| New 5-grade | 5 | 1 | I | 30 | 26 | 86.7 |
| New 5-grade | 5 | 2 | II | 30 | 25 | 83.3 |
| New 5-grade | 5 | 3 | III | 30 | 26 | 86.7 |
| Traditional 3-grade | Ⅰ | 1 | I | 30 | 27 | 90 |
| Traditional 3-grade | Ⅰ | 2 | II | 30 | 27 | 90 |
| Traditional 3-grade | Ⅰ | 3 | III | 30 | 28 | 93.3 |
| Traditional 3-grade | Ⅱ | 1 | I | 30 | 26 | 86.7 |
| Traditional 3-grade | Ⅱ | 2 | II | 30 | 27 | 90 |
| Traditional 3-grade | Ⅱ | 3 | III | 30 | 26 | 86.7 |
| Traditional 3-grade | Ⅲ | 1 | I | 30 | 26 | 86.7 |
| Traditional 3-grade | Ⅲ | 2 | II | 30 | 25 | 83.3 |
| Traditional 3-grade | Ⅲ | 3 | III | 30 | 25 | 83.3 |
| **Total** |  | **24 plots** |  | **720** |  | **90.1** |
| **Note:** Each plot initially contained 30 seedlings at transplanting (Day 0). Survival was assessed at 180 days post-transplant. Survival rate = (Surviving seedlings / 30) × 100%. | | | | | | |
