# Supplementary material for: Research on seedling grading of Platycladus orientalis based on fuzzy C-means clustering algorithm improved by genetic sparrow family search optimization
Source: Front Plant Sci. 2026 Sep 3;17:1929618. doi: 10.3389/fpls.2026.1929618 (PMC13582205; doi:10.3389/fpls.2026.1929618)
Supplement: Supplementary file 2 [file Table2.docx]

| **Table S2: Statistical evidence supporting the merger of Clusters 4 and 5 into a single grade** | | | | | | | | | | | | | | | | | |
| --- | --- | --- | --- | --- | --- | --- | --- | --- | --- | --- | --- | --- | --- | --- | --- | --- | --- |
| **Part A:** Multivariate tests on five-dimensional cluster centroids (Cluster 4 vs. Cluster 5) | | | | | | | | | | | | | | | | | |
| **Statistic** | | | | | **Value** | | | | **Critical value (α = 0.05)** | | | | | **Decision** | | | |
| Hotelling's T² | | | | | 1.40 | | | | 11.88 | | | | | Not significant | | | |
| F(5, 167) | | | | | 0.27 | | | | 2.27 | | | | | p = 0.931 | | | |
| Mahalanobis D² | | | | | 0.032 | | | | 0.271 | | | | | D² << D²_crit | | | |
| η² (effect size) | | | | | 0.008 | | | | — | | | | | Negligible | | | |
| Power (observed effect) | | | | | 0.12 | | | | — | | | | | Low power for trivial effect | | | |
| Power (medium f² = 0.15) | | | | | 1.00 | | | | — | | | | | Sufficient | | | |
| **Note:** n4= 81, n5= 96. All statistics computed from Table 7 cluster centers and SDs, using within-cluster SD = center SD × √n and diagonal covariance assumption. The observed effect (T² = 1.40) is far below the minimum detectable T² = 13.26 for 80% power, confirming the two clusters are genuinely indistinguishable rather than failing to detect a real difference. | | | | | | | | | | | | | | | | | |
| **Part B:** Per-feature Cohen's d (Cluster 4 vs. Cluster 5) | | | | | | | | | | | | | | | | | |
| **Feature** | | **Cluster 4  (mean±SD)** | | | **Cluster 5  (mean±SD)** | | **Difference** | | | **Cohen's d** | | **95% CI** | | | | **Interpretation** | |
| Stem diameter (mm) | | 5.19±0.14 | | | 5.07±0.13 | | +0.12 | | | +0.10 | | [-0.20, +0.39] | | | | Negligible | |
| Plant height (mm) | | 618.9±8.9 | | | 612.9±9.4 | | +6.0 | | | +0.07 | | [-0.23, +0.37] | | | | Negligible | |
| Branch number | | 16.9±0.8 | | | 17.3±0.9 | | −0.4 | | | −0.05 | | [-0.35, +0.25] | | | | Negligible | |
| θ @ 4 kHz (°) | | −24.33±0.43 | | | −23.81±0.52 | | −0.52 | | | −0.11 | | [-0.41, +0.18] | | | | Negligible | |
| tan δ @ 400 kHz | | 0.358±0.019 | | | 0.348±0.021 | | +0.010 | | | +0.05 | | [-0.24, +0.35] | | | | Negligible | |
| **Note:** Criterion: \|d\| < 0.20 for all features → no meaningful difference. All five features satisfied this criterion (max \|d\| = 0.11). | | | | | | | | | | | | | | | | | |
| **Part C:** Post-merger field validation (five-grade system, from Table 10) | | | | | | | | | | | | | | | | | |
| **Adjacent grade pair** | | | | **Survival rate diff. (%)** | | | | **Cohen's d** | | | | | **p-value** | | | | |
| Grade 1 vs. Grade 2 | | | | 12.8 | | | | 1.39 | | | | | 0.023 | | | | |
| Grade 2 vs. Grade 3 | | | | 8.5 | | | | 1.12 | | | | | 0.017 | | | | |
| Grade 3 vs. Grade 4 | | | | 5.2 | | | | 0.84 | | | | | 0.031 | | | | |
| Grade 4 vs. Grade 5 | | | | 3.8 | | | | 0.91 | | | | | 0.014 | | | | |
| **Note:** After merging Clusters 4 and 5 into Grade 4, the five-grade system showed significant separation between all adjacent grades (all p < 0.05, d = 0.84–1.39, large effects). Growth increment comparisons yielded d = 1.25–2.38. Pre-merger field comparison between Clusters 4 and 5 showed non-significant differences for all five field metrics (survival rate, height increment, stem diameter increment, branch number increment, total biomass; all p > 0.05, \|d\| < 0.20), confirming field-performance equivalence prior to merging. | | | | | | | | | | | | | | | | | |
| **Part D:** Contrast with all other adjacent cluster pairs | | | | | | | | | | | | | | | | | |
| **Cluster pair** | **n (A, B)** | | **Hotelling's T²** | | | **F(df_1_, df_2_)** | **p-value** | | **Mahalanobis D²** | | **max \|d\|** | | | | **T² ratio vs. C4vsC5** | | **Merge?** |
| C1 vs. C2 | 65, 81 | | 55.94 | | | 10.57 (5, 136) | < 0.001 | | 1.551 | | 0.71 | | | | 40× | | No |
| C2 vs. C3 | 81, 105 | | 35.02 | | | 6.70 (5, 176) | < 0.001 | | 0.766 | | 0.52 | | | | 25× | | No |
| C3 vs. C4 | 105, 81 | | 215.24 | | | 41.18 (5, 176) | < 0.001 | | 4.707 | | 1.84 | | | | 154× | | No |
| **C4 vs. C5** | **81, 96** | | **1.40** | | | **0.27 (5, 167)** | **0.931** | | **0.032** | | **0.11** | | | | **1×** | | **Yes** |
| C5 vs. C6 | 96, 72 | | 137.12 | | | 26.10 (5, 158) | < 0.001 | | 3.333 | | 1.17 | | | | 98× | | No |
| **Note:** Sample sizes: n1= 65, n2= 81, n3= 105, n4= 81, n5= 96, n6= 72 (N = 500). All other adjacent pairs showed highly significant separation (T² = 35.0–215.2, all p < 0.001, max \|d\| = 0.52–1.84), with T² values 25–154 times that of C4 vs. C5. This confirms that the merging decision is specific to Clusters 4 and 5 and not a general artifact of the protocol. | | | | | | | | | | | | | | | | | |

# CRediT authorship contribution statement

**Yang Liu:** Conceptualization, Methodology, Writing-Original draft. **Juan Zhou:** Investigation, Writing - Review & Editing. **Juan Wang**: Visualization. **Bao Di:** Data curation. **Chao Wang:** Software. **ZhenHui Ren:** Formal analysis, Validation, Supervision.

# Declaration of Competing Interest

We declare that none of the work contained in this manuscript is published in any language or currently under consideration at any other journal, and there are no conflicts of interest to declare. All authors have contributed to, read, and approved this submitted manuscript in its current form.

# Acknowledgements

The authors are grateful for the financial support from the projects of the Hebei Provincial Department of Education and S&T Program of Baoding.

# Funding Declaration

This research was funded by the projects of the Hebei Provincial Department of Education and S&T Program of Baoding.

# Clinical trial number

Not applicable.

# Ethics, Consent to Participate, and Consent to Publish declarations

Not applicable.

**Availability of Data and Materials**

All data generated or analyzed during the current study are available from the corresponding author upon reasonable request, via email: renzh68@163.com.

**References:**

[1] Zhang S., et al. Studies on Mechanisms of Molecular Response to Drought and Natural Low Temperature Stress in Platycladus orientalis[D]. Northwest Agriculture and Forestry University, 2017.

[2] L Li, X Song, D Feng, et al. Impacts of climate change on soil desiccation in planted forests with different tree ages: A case study in the Loess Plateau of China[J]. Ecol. Indic., 2023, 148: 110073.

[3] H Dou, H Sun, X Feng, et al. Full-Length transcriptome assembly of Platycladus orientalis root integrated with RNA-Seq to identify genes in response to root pruning[J]. Forests, 2024, 15(7): 1232.

[4] E Chang, W Guo, Y Dong, et al. Metabolic profiling reveals key metabolites regulating adventitious root formation in ancient Platycladus orientalis cuttings[J]. Front. Plant Sci., 2023, 14: 1192371.

[5] Ou J.D., et al. Seedling Quality Grading of Cunninghamia konishii in Fujian[J]. J. Southwest For. Univ. (Nat. Sci.), 2018, 38(02): 172-176.

[6] Y Liu, J Qian, X Yang, et al. Study on measurement method for apple root morphological parameters based on Labview[J]. Plant Methods, 2019, 15(1): 149.

[7] Y Hirano, M Dannoura, K Aono, et al. Limiting factors in the detection of tree roots using ground-penetrating radar[J]. Plant Soil, 2009, 319(1-2): 15-24.

[8] Y Cao, T Repo, R Silvennoinen, et al. Analysis of the willow root system by electrical impedance spectroscopy[J]. J. Exp. Bot., 2010, 62(1): 351-358.

[9] I Cseresnyés, K Rajkai, E Vozáry. Role of phase angle measurement in electrical impedance spectroscopy[J]. Int. Agrophys., 2013, 27(4): 377-383.

[10] Y Liu, D Li, J Qian, et al. Electrical impedance spectroscopy (EIS) in plant roots research: A review[J]. Plant Methods, 2021, 17(1): 118.

[11] Chen S., Du T.S., Wang S. Delineating Management Zones in Maize Field Based on Fuzzy C-Means Algorithm[J]. Trans. Chin. Soc. Agric. Mach., 2020, 50(11): 293-300.

[12] T Repo, A Korhonen, T Lehto, et al. Assessment of frost damage in mycorrhizal and non-mycorrhizal roots of Scots pine seedlings using classification analysis of their electrical impedance spectra[J]. Trees, 2016, 30(2): 483-495.

[13] Li F., et al. Nondestructive Detection of Apple Mouldy Core Based on Bioimpedance Properties[J]. Food Sci., 2013, 34(18): 197-202.

[14] Cai C., et al. Non-destructive detection of freshness grade for apple fruit based on bio-impedance properties[J]. Trans. Chin. Soc. Agric. Mach., 2013, 44(2): 147-152.

[15] S Kumar, K Ziauddin. Hybrid Features based Multimodal Biometric Identification using FCM and Support Vector Machine Classifier[J]. Wesleyan Theol. J., 2021, 13(68): 79-92.

[16] B Cardone, F D Martino, S Senatore. Improving the emotion-based classification by exploiting the fuzzy entropy in FCM clustering[J]. Int. J. Intell. Syst., 2021, 36(11): 6944-6967.

[17] R Aarthi, D Sivakumar. An enhanced agricultural data mining technique for dynamic soil texture prediction[J]. Procedia Comput. Sci., 2020, 171: 2770-2778.

[18] B Di, J Luoranen, T Lehto, et al. Biophysical changes in the roots of Scots pine seedlings during cold acclimation and after frost damage[J]. For. Ecol. Manage., 2019, 431: 63-72.

[19] Guo L. Study on the impedance spectroscopy during drought resistant of Platycladus orientalis[D]. Beijing Forestry University, 2008: 59.

[20] T Repo, J Laukkanen, R Silvennoinen. Measurement of the tree root growth using electrical impedance spectroscopy[J]. Silva Fenn., 2005, 39(2): 159-168.

[21] H Ozier-Lafontaine, T Bajazet. Analysis of root growth by impedance spectroscopy (EIS)[J]. Plant Soil, 2005, 277(1-2): 299-313.

[22] T Repo, A Korhonen, M Laukkanen, et al. Detecting mycorrhizal colonisation in Scots pine roots using electrical impedance spectra[J]. Biosyst. Eng., 2014, 121: 139-149.

[23] Yuan S. Application of Clustering Algorithm Based on Attribute Weighting in Bank Customer Segmentation[D]. Lanzhou University of Finance and Economics, 2020: 74.

[24] Liu C.S., Xu Q.L. Fuzzy C-means clustering algorithm based on density peak value optimization[J]. Comput. Eng. Appl., 2018, 54(14): 153-157.

[25] J Xue, B Shen. A novel swarm intelligence optimization approach: Sparrow search algorithm[J]. Syst. Sci. Control Eng., 2020, 8(1): 22-34.

[26] X Y Zhang, Z C Dong, M A Hong-Liang. Study on Optimization Operation of Xiaolangdi Reservoir Based on Improved Multi-objective Genetic Algorithm[J]. Water Resour. Power, 2017, 35(01): 65-68.

[27] Wang B., et al. Hybrid multi-objective grasshopper optimization algorithm based on fusion of multiple strategies[J]. J. Comput. Appl., 2020, 40(9): 2670-2676.

[28] Q Liang, B Chen, H Wu, et al. A novel modified sparrow search algorithm with application in side lobe level reduction of linear antenna array[J]. Wirel. Commun. Mob. Comput., 2021, 2021: 1-25.

[29] R K Raigar, R Upadhyay, H N Mishra. Storage quality assessment of shelled peanuts using non-destructive electronic nose combined with fuzzy logic approach[J]. Postharvest Biol. Technol., 2017, 132: 43-50.

[30] Liu Y.Z. Application of Principal Component Analysis and Clustering in Science and Technology Data Analysis[D]. Jilin University, 2016: 57.

[31] Grossnickle S., Macdonald J. Seedling Quality: History, Application, and Plant Attributes[J]. Forests, 2018, 9(5): 283.
[32] Liu Z.H., Cui Y.H., Zheng F., Liu Y.M., Liu Y., Ge Y.L., Wang A.F. Effects of waterlogging in summer on electrical impedance characteristics, growth and physiology of roots in Pinus bungeana seedlings[J]. Acta Ecol. Sin., 2024, 44(18): 8208-8218.

[33] Hurlbert,S.H. Pseudoreplication and the Design of Ecological Field Experiments[J]. Ecological Monographs, 1984, 54(2): 187–211.

[34] Li G , Du S , Wen Z .Mapping the climatic suitable habitat of oriental arborvitae (Platycladus orientalis) for introduction and cultivation at a global scale[J].Scientific Reports, 2016, 6:30009.

[35] Benjamini Y , Hochberg Y .Controlling the False Discovery Rate: A Practical and Powerful Approach to Multiple Testing[J]. Journal of the Royal Statistical Society. Series B: Methodological, 1995, 57(1):289-300.

[36] Jolliffe I T. Discarding variables in a principal component analysis. I: Artificial data[J]. Journal of the Royal Statistical Society Series C: Applied Statistics, 1972, 21(2): 160-173.
[37] Bezdek J C .Pattern Recognition With Fuzzy Objective Function Algorithms[J].Plenum Press, 1981.

[38] Yao K, Tu C, Zhang A, et al. Distinguishing drought resistance strategies and identifying indicator traits of Platycladus orientalis and Broussonetia papyrifera[J]. Frontiers in Plant Science, 2025, 16: 1644756.
